# First multi-locus sequence typing scheme for *Arcobacter *spp.

**DOI:** 10.1186/1471-2180-9-196

**Published:** 2009-09-14

**Authors:** William G Miller, Irene V Wesley, Stephen LW On, Kurt Houf, Francis Mégraud, Guilin Wang, Emma Yee, Apichai Srijan, Carl J Mason

**Affiliations:** 1 Produce Safety and Microbiology Research Unit, Agricultural Research Service, U.S. Department of Agriculture, Albany, CA 94710, USA; 2 Agricultural Research Service, U.S. Department of Agriculture, National Animal Disease Center, Ames, IA 50010, USA; 3 Food Safety Programme, Institute of Environmental Science & Research Limited, Christchurch Science Centre, Christchurch, New Zealand; 4 Faculty of Veterinary Medicine, Department of Veterinary Public Health and Food Safety, Ghent University, Merelbeke, Belgium; 5 Laboratoire de Bactériologie, Université Victor Segalen, Bordeaux, France; 6 Department of Enteric Diseases, AFRIMS, Bangkok, Thailand

## Abstract

**Background:**

*Arcobacter *spp. are a common contaminant of food and water, and some species, primarily *A. butzleri *and *A. cryaerophilus*, have been isolated increasingly from human diarrheal stool samples. Here, we describe the first *Arcobacter *multilocus sequence typing (MLST) method for *A. butzleri*, *A. cryaerophilus*, *A. skirrowii, A. cibarius *and *A. thereius*.

**Results:**

A sample set of 374 arcobacters, including 275 *A. butzleri*, 72 *A. cryaerophilus*, 15 *A. skirrowii *and 8 *A. cibarius *isolates from a wide variety of geographic locations and sources, was typed in this study. Additionally, this sample set contained four strains representing a new *Arcobacter *species, *A. thereius*. The seven loci used in the four-species *Arcobacter *MLST method are the same as those employed previously in *C. jejuni*, *C. coli*, *C. helveticus *and *C. fetus *(i.e. *aspA*, *atpA*(*uncA*), *glnA*, *gltA*, *glyA, pgm *and *tkt*). A large number of alleles were identified at each locus with the majority of isolates containing a unique sequence type. All *Arcobacter *isolates typed in this study contain two *glyA *genes, one linked to *lysS *(*glyA1*) and the other linked to *ada *(*glyA2*). *glyA1 *was incorporated into the *Arcobacter *MLST method while *glyA2 *was not because it did not increase substantially the level of discrimination.

**Conclusion:**

No association of MLST alleles or sequence types with host or geographical source was observed with this sample set. Nevertheless, the large number of identified alleles and sequence types indicate that this MLST method will prove useful in both *Arcobacter *strain discrimination and in epidemiological studies of sporadic *Arcobacter*-related gastroenteritis. A new *Arcobacter *MLST database was created http://pubmlst.org/arcobacter/; allele and ST data generated in this study were deposited in this database and are available online.

## Background

The genus *Arcobacter *is a member of the Gram-negative, ε-Proteobacterial subdivision. The majority of isolated arcobacters belong to one of three species: *Arcobacter butzleri*, *A. cryaerophilus *or *A. skirrowii*. Additional members of this taxon include: *A. cibarius*, isolated from broiler carcasses [1]; *A. nitrofigilis*, a nitrogen-fixing organism isolated originally from estuarine plant roots [2]; *A. halophilus*, isolated from a hypersaline lagoon [3]; *Candidatus *Arcobacter sulfidicus, a sulfide-oxidizing marine organism [4]; *A. mytili *sp. nov., isolated from mussels [5]; *A. thereius *sp. nov, isolated from pigs and ducks [6] and *A. marinus *sp. nov [7]. *Arcobacter butzleri*, *A. cryaerophilus*, *A. skirrowii *and *A. cibarius *have been isolated often from both animals [8-10] and food sources [10-13], water and agricultural runoff [10,14-16], and domestic pets [17].

The prevalence of arcobacters in food, raw milk and water would suggest a potential for food- or water-borne *Arcobacter*-associated human illness. *Arcobacter *spp., primarily *A. butzleri *and *A. cryaerophilus*, have been isolated from human diarrheal stool samples [18-22]. However, no direct connection between consumption of *Arcobacter*-contaminated food or water and human illness has been established, although it is likely that transmission of arcobacters occur via these routes. *Arcobacter *spp. have been isolated also from the stools of healthy humans [20,23]. Thus, while host predispositions such as age and immune status may play a role, it is possible that some *A. butzleri *and *A. cryaerophilus *strains are non-pathogenic and are human commensals. The presence of a subset of non-pathogenic strains alongside pathogenic strains within *A. butzleri*, *A. cryaerophilus *and perhaps the other food- and water-associated *Arcobacter *species, such as *A. skirrowii *and *A. cibarius*, would indicate a need for an accurate typing method to distinguish human-pathogenic and human-commensal arcobacters. *Arcobacter *typing methodology would also be useful in studies of transmission routes and source tracking during outbreak and extended epidemiological investigations. Typing of *Arcobacter *strains using such methods as enterobacterial repetitive intergenic consensus (ERIC)-PCR, pulsed field gel electrophoresis (PFGE) and amplified fragment length polymorphism (AFLP) analysis has been reported (reviewed in [10]).

Multilocus sequence typing (MLST), a typing method based on partial, yet defined, sequence information at seven housekeeping loci, was developed first within the ε-Proteobacteria for *C. jejuni *[24]. It has proven useful for strain characterization, lineage identification and *C. jejuni *epidemiology (reviewed in [25]). Within *Campylobacter*, MLST methods are available also for *C. coli *[26,27], *C. lari *[27], *C. upsaliensis *[27], *C. helveticus *[27], *C. fetus *[28] and *C. insulaenigrae *[29]. The existence of multiple MLST methods within a genus provides insights into much broader areas, such as the degree of horizontal gene transfer between species and bacterial evolution and speciation within a genus; MLST can provide additional, clarifying genotypic information for a novel or potentially novel species [29]. Development of the non-*jejuni Campylobacter *MLST methods was assisted by the availability of draft *C. coli*, *C. lari *and *C. upsaliensis *genomes [30]. Construction of degenerate primer sets, based on alignments of these genome sequences at the seven MLST loci, permitted extension of the MLST methods into two species, *C. insulaenigrae *and *C. helveticus*, for which no genomic information existed [27,29]. Similarly, the existence of the recently completed *A. butzleri *strain RM4018 genome [31], as well as a draft genome of *A. halophilus *strain LA31B (Miller et al., unpublished data), provided useful information for the development of an MLST method suitable for typing of *Arcobacter *species.

Here, we describe a new MLST method for multiple *Arcobacter *species, including the three most frequently-isolated *Arcobacter *spp., *A. butzleri*, *A. cryaerophilus *and *A. skirrowii*. The *Arcobacter *MLST gene set is identical to the *C. jejuni *gene set (i.e. *aspA*, *atpA*(*uncA*), *glnA*, *gltA*, *glyA*, *pgm *and *tkt*), permitting phylogenetic comparison of data across the two genera. A sample set of 374 isolates of diverse geographic origin and source was typed in this study. Almost 300 sequence types and 1176 alleles across seven loci were identified.

## Results and Discussion

### Design of the *Arcobacter *multilocus sequence typing method

In order to optimize cross-species and cross-genus comparisons, the four genes (*atpA*(*uncA*), *glnA*, *glyA *and *tkt*) common to the *Campylobacter *MLST methods described previously [24,26-29], together with the three additional loci (*aspA*, *gltA *and *pgm*) present within the *C. jejuni *method [24], were targeted in the *Arcobacter *MLST method. For optimal phylogenetic comparison, the same allelic endpoints were considered. Development of the *Arcobacter *MLST method was assisted by the concurrent completion of the *A. butzleri *strain RM4018 genome sequence [31]. Gene sequences for the seven *C. jejuni *MLST loci were extracted, where applicable, from the existing *Arcobacter *and thermotolerant *Campylobacter *genome sequences, and aligned. Degenerate primers, situated approximately 300 bp upstream and downstream from the allelic endpoints, were designed and 94 *Arcobacter *strains (i.e. 69 *A. butzleri*, 21 *A. cryaerophilus *and 4 *A. skirrowii*) were amplified and sequenced. Sequence information from this sample set was aligned and used to construct the *butzleri*-specific primers listed in Table S1 [see additional file 1]. For the non-*butzleri *species, some loci did not amplify efficiently, using primers based on the *Campylobacter*/*Arcobacter *alignments. For these loci, improved primer pairs were constructed by incorporating sequences from the draft *A. halophilus *genome (Miller et al., unpublished data) into the *Campylobacter*/*Arcobacter *alignments. These improved primer pairs efficiently amplified the seven MLST loci (i.e. *aspA*, *atpA*, *glnA*, *gltA*, *glyA*, *pgm *and *tkt*) of *A. cryaerophilus *and *A. skirrowii *[see additional file 1 - Table S1].

Initial typing of the *Arcobacter *sample set at the *glyA *locus resulted in mixed sequencing reads for some strains, suggesting that at least two *glyA *genes might be present. The presence of multiple *glyA *genes was confirmed later upon completion of the *A. butzleri *strain RM4018 genome [31]. In this strain, two nearly-identical, complete *glyA *genes are present in the genome, one (*glyA1*) linked to *lysS *and the other (*glyA2*) to *ada*. Therefore, to eliminate generation of mixed traces, amplification primers were designed within the *lysS *and *ada *genes. PCRs using the *lysS *and *glyA *reverse primers amplified specifically *glyA1 *and PCRs using the *ada *and *glyA *forward primers amplified specifically *glyA2*. All *Arcobacter *isolates typed in this study contained at least two *glyA *genes, suggesting that the presence of multiple *glyA *genes is an unusual feature common to the genus. The *glyA *locus in other *Campylobacter *MLST methods is also linked to *lysS*. For this reason, and for the fact that the *glyA2 *locus is less discriminatory than *glyA1 *(see below), the *lysS*-linked *glyA1 *locus was incorporated into the *Arcobacter *typing method.

### *Arcobacter *strain characterization

To address the ability of the *Arcobacter *MLST method to amplify successfully as many *A. butzleri *strains as possible, we wanted a large sample set with broad geographic origins and sources. A description of the *Arcobacter *isolates by geographic origin and source is listed in Tables 1 and 2. A total of 275 *A. butzleri *isolates were typed, as well as an additional 99 isolates representing other *Arcobacter *species, e.g. *A. cryaerophilus *and *A. skirrowii *[see additional file 2 - Table S2]. All 366 *A. butzleri*, *A. cryaerophilus*, *A. skirrowii *and *A. thereius *isolates amplified and sequenced successfully with one or more of the primer pairs listed in Table S1 [see additional file 1]. *Arcobacter cibarius *demonstrated variable *tkt *amplification results, i.e. weak amplification of some loci with each primer pair and no primer pair amplifying all loci [see additional file 1 - Table S1].

**Table 1:** Geographic origin of the *Arcobacter *strains typed in this study.

	*A. butzleri*	*A. cryaerophilus*	*A. skirrowii*	*A. thereius*	*A. cibarius*
Belgium	4	1	1	-----	8
Canada	2	-----	2	-----	-----
Denmark	6	1	5	3	-----
France	14	1	-----	-----	-----
Germany	1	-----	-----	-----	-----
Greece	1	-----	-----	-----	-----
Ireland/N. Ireland	4	20	2	-----	-----
Netherlands	1	-----	-----	-----	-----
Nigeria	9	-----	-----	-----	-----
South Africa	2	-----	-----	-----	-----
Sweden	4	-----	-----	-----	-----
Thailand	118	-----	-----	-----	-----
Turkey	10	-----	-----	-----	-----
UK	3	-----	3	-----	-----
U.S.A.	65	10	1	-----	-----
Vietnam	15	-----	-----	-----	-----
Unknown	16	39	1	1	-----

Total	275	72	15	4	8

**Table 2:** Source of the *Arcobacter *strains typed in this study.

	*A. butzleri*	*A. cryaerophilus*	*A. skirrowii*	*A. thereius*	*A. cibarius*
Cattle	3	14	4	-----	-----
Beef	14	-----	-----	-----	-----
Lamb/Sheep	4	-----	1	-----	-----
Chicken	60	-----	2	-----	8
Poultry	15	4	-----	-----	-----
Eggs	1	-----	-----	-----	-----
Swine	16	45	6	3	-----
Pork	27	-----	-----	-----	-----
Turkey	18	1	-----	-----	-----
Duck	2	1	2	1	-----
Fish	3	-----	-----	-----	-----
Shrimp	1	-----	-----	-----	-----
Squid	3	-----	-----	-----	-----
Horse	1	2	-----	-----	-----
Primate	3	-----	-----	-----	-----
Human	102	4	-----	-----	-----
Unknown	2	1	-----	-----	-----

Total	275	72	15	4	8

### Genetic diversity of the *Arcobacter *MLST loci

A large number of *Arcobacter *MLST alleles and sequence types (STs) were identified in this study (Table 3). Allelic density (i.e. no. alleles/no. strains) ranged from approximately 30% (111/374) at the *glnA *locus to 63% (236/374) at the *glyA1 *locus. The high density of alleles translated also into a large number of STs (Table 3). Among the 275 *A. butzleri *isolates characterized in this study, 208 STs were identified. In fact, among all of the *Arcobacter *STs, no more than five strains were determined to possess the same ST and 228 of 374 strains (61%) contained unique STs. A large percentage of variable sites were identified at all of the *Arcobacter *MLST loci (Table 4). *Arcobacter cryaerophilus *and *A. skirrowii *strains contained the highest number of variable sites per locus, relative to the number of alleles identified, and the largest number of variable sites for all species occurred at the *glyA *and/or *pgm *loci.

**Table 3:** *Arcobacter *alleles and sequence types.

		Alleles									
			
	Strains	*aspA*	*atpA*	*glnA*	*gltA*	Total *gly*	*glyA1*	*glyA2*	*pgm*	*tkt*	STs
*A. butzleri*	275	81	69	51	66	217	162	150	127	90	208
*A. cryaerophilus*	72	49	35	44	38	92	56	55	51	52	59
*A. skirrowii*	15	12	12	12	8	17	13	10	9	7	14
*A. thereius*	4	3	3	3	4	5	3	3	2	2	4
*A. cibarius*	8	1	1	1	3	3	2	2	2	4	5

TOTAL	374	146	120	111	119	334	236	220	191	155	290

**Table 4:** Diversity of *Arcobacter *alleles and sequence types.

		*aspA*	*atpA*	*glnA*	*gltA*	*glyA1*	*glyA2*	*pgm*	*tkt*
*A. butzleri*	VS^a^	58	47	45	36	72	58	83	66
	*d*_*n*_/*d*_*s*_^b^	0.016	0.093	0.024	0.000	0.087	0.085	0.024	0.032
*A. cryaerophilus*	VS	91	66	100	70	140	143	78	73
	*d*_*n*_/*d*_*s*_	0.038	0.053	0.051	0.058	0.125	0.135	0.050	0.046
*A. skirrowii*	VS	30^c^	22	66^c^	11	75	69	13	35
	*d*_*n*_/*d*_*s*_	0.057	0.030	0.142	0.118	0.128	0.114	0.145	0.181

**a**. Variable sites

**b**. Ratio of non-synonymous to synonymous sites.

**c**. An additional 53 and 37 variable sites are present within the *aspA *and *glnA *loci, respectively, when *A. skirrowii *ST-243 is included in the calculations.

The identification of MLST alleles associated with particular food animal sources was first described in *C. coli *[32]. However, analysis of the *A. butzleri *and *A. cryaerophilus *MLST alleles and STs revealed no apparent host-association. Additionally, phylogenetic analysis of *A. butzleri *and *A. cryaerophilus *alleles and STs did not identify any clusters or groups associated with geographic origin

The *d*_*n*_/*d*_*s *_ratio (i.e., the ratio of substitution rates at non-synonymous and synonymous sites) was substantially < 1 for all of the MLST loci characterized in this study (Table 4), ranging from 0.000 at *A. butzleri gltA *to 0.181 at *A. skirrowii tkt*. These low values for the *Arcobacter *MLST loci are consistent with those described previously for *Campylobacter *[24,27,29], indicating that those loci in both genera are not subject to positive selection.

The presence of a large number of MLST alleles within the *Arcobacter *sample set might indicate that the *Arcobacter *MLST alleles are genetically unstable, prone to change either by accumulation of point mutations or horizontal gene transfer. Four *A. butzleri *type strain isolates, obtained from different labs and including the genome sequence strain RM4018, were typed in this study. In addition, 17 related strains, isolated after passage of the *A. butzleri *type strain through swine, were also typed. As expected, all 21 strains were the same sequence type, ST-1, and contained the same *glyA2 *allele (data not shown), suggesting that *A. butzleri *STs are relatively stable, even after passage through a food animal.

### Association of *Arcobacter *alleles and STs with species and subgroups

**Figure 1:**
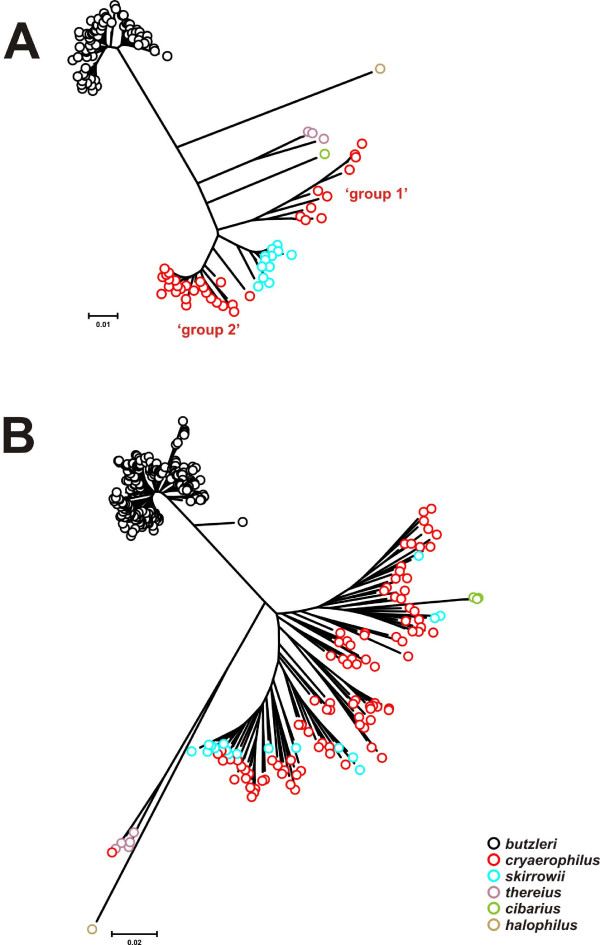
**Dendrograms of *Arcobacter atpA *and *glyA *alleles**. A: *atpA*; B: *glyA*. The dendrograms were constructed using the neighbor-joining algorithm and the Kimura two-parameter distance estimation method. The scale bars represent substitutions per site. The *A. halophilus *strain LA31B *atpA *and *glyA *sequences were extracted from the draft *A. halophilus *genome. Note the presence of a putative laterally-transferred allele within the *A. thereius glyA *cluster.

Within each of the *aspA*, *atpA*, *glnA*, *gltA*, *pgm *and *tkt *loci, phylogenetically discrete clusters were identified that associated with species (data not shown). An example is illustrated in Figure 1A for the *atp *locus, showing that the *A. butzleri*, *A. skirrowii*, *A. thereius *and *A. cryaerophilus *alleles form distinct groups. However, for the latter species two separate clusters, termed here 'group 1' and 'group 2' were observed. Two phylogenetically-distinct clusters of *A. cryaerophilus *alleles were identified also at the *glnA*, *gltA*, *pgm *and *tkt *loci [see additional file 2 - Table S2], but not at the *aspA *locus that formed only one cluster. The existence of species-associated clustering at these six loci permits tentative identification of lateral transfer events. These events were not observed in *A. butzleri *because no alleles related phylogenetically to other species were identified, however, alleles related phylogenetically to those identified in *A. butzleri *were identified within *A. cibarius *and *A. skirrowii *(i.e. *tkt-90*, *tkt-91*, *aspA-73 *and *glnA-1*). Similarly, *A. skirrowii *alleles were identified within *A. cryaerophilus *and *A. thereius (*e.g. *aspA-125 *and *glnA-95)*, and an *A. thereius *allele was identified in *A. cryaerophilus *(*glyA-306*; see Figure 1B). Lateral transfer events identified by MLST have been reported previously [27,32].

Clustering of the *glyA *alleles (including alleles at both *glyA *genes) is noticeably different from clustering at the other six loci (Figure 1B). Here, as at the other six loci, the *A. butzleri *and *A. thereius glyA *alleles form separate clusters distinct from the alleles of the other characterized arcobacters. However, the *glyA *alleles of *A. cryaerophilus *and *A. skirrowii *are indistinguishable phylogenetically, with the *A. cibarius glyA *alleles forming a deep branch within the *A. cryaerophilus*/*A. skirrowii *cluster. Additionally, the *A. cryaerophilus*/*A. skirrowii glyA *cluster is highly divergent, relative to the *A. cryaerophilus *and *A. skirrowii *clusters at the other MLST loci.

**Figure 2:**
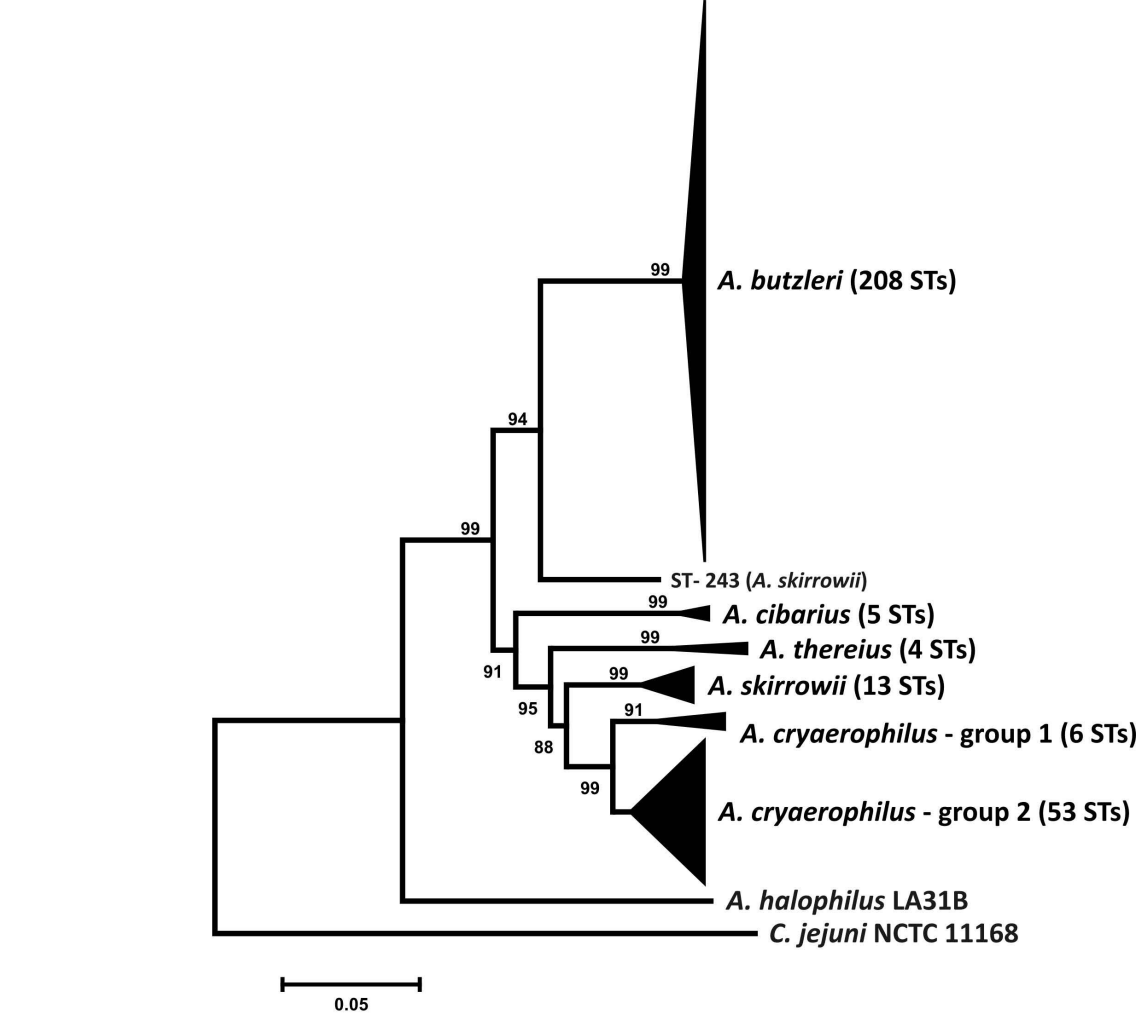
**Condensed dendrogram of unique *Arcobacter *STs**. For each unique ST, the profile allele sequences were extracted and concatenated. The concatenated allele sequences were aligned using CLUSTAL X (ver. 2.0.5). The dendrogram was constructed using the neighbor-joining algorithm and the Kimura two-parameter distance estimation method. Bootstrap values of >75%, generated from 500 replicates, are shown at the nodes. The scale bar represents substitutions per site. The tree is rooted to *C. jejuni *strain NCTC 11168. The *A. halophilus *strain LA31B concatenated sequence was extracted from the draft *A. halophilus *genome. 'Group 1' *A. cryaerophilus *sequence types include: ST-209, ST-220, ST-221, ST-231, ST-232 and ST-270.

Phylogenetic analysis of the *Arcobacter *STs, following CLUSTAL alignment of the concatenated allele sequences for each unique profile, indicated that these STs clustered also by species (Figure 2). *Arcobacter thereius *profiles formed a clade distinct from *A. skirrowii *and the other *Arcobacter *species, providing additional evidence that the strains within this clade are exemplars of a novel *Arcobacter *species. Two groups of *A. cryaerophilus *profiles were observed: 'group 1' and 'group 2' profiles were composed primarily of 'group 1' and 'group 2' MLST alleles, respectively. Based on SDS-PAGE analysis of whole-cell protein extracts and 16S restriction fragment length polymorphism analysis, two subgroups within *A. cryaerophilus *were identified by Kiehlbauch et al. and Vandamme et al. [33,34]. These *A. cryaerophilus *subgroups differ also in their fatty-acid composition, specifically in the amounts of 16:1 *cis *9 and 16:1 *trans *9 fatty acids [33], and in their AFLP profiles [35], although they are not differentiated by DNA:DNA hybridization analysis [33]. Only 7 of the 72 *A. cryaerophilus *strains in this study were characterized previously at the subgroup level by either AFLP or whole protein profiling [see additional file 2 - Table S2]. However, the subgroup identities of these strains did not correlate well with the MLST groups. Considering these results, it is possible that the *cryaerophilus *subgroups identified by Vandamme et al. [33] are not analogous to the MLST groups identified here, although additional investigations will be necessary to resolve this issue.

### The *Arcobacter glyA1 *and *glyA2 *loci

As described above, *Arcobacter *strains contain two unlinked *glyA *genes in their genomes. The *ada*-linked *glyA2 *alleles are less discriminatory than the *lysS*-linked *glyA1 *alleles: incorporation of *glyA2 *into the typing scheme in place of *glyA1 *would result in 197 STs for *A butzleri*, instead of 208, and 58 STs for *A. cryaerophilus*, instead of 59. Therefore, this reduced level of discrimination was one of the reasons why the *ada*-linked *glyA2 *locus was not incorporated into the *Arcobacter *MLST method. Additionally, inclusion of both *glyA *loci in the *Arcobacter *MLST method, thus creating an eight-locus typing scheme, would not increase significantly the discriminatory power of the seven locus method. A large number of STs contain different *glyA1 *and *glyA2 *alleles: for example, the *A. butzleri *genome sequence strain RM4018 contains the *glyA-1 *allele at the *glyA1 *locus and *glyA-142 *at the *glyA2 *locus [see additional file 2 - Table S2].

The presence of two highly-similar *glyA *loci is an unusual feature of the *Arcobacter *genomes and multiple copy genes are not generally members of MLST schemes. However, the data suggest that despite the presence of two *glyA *loci within every strain, the *Arcobacter glyA *loci are remarkably stable. There is no compelling evidence in this study (with the possible exception of ST-240) of gene conversion events between the two *glyA *genes (manifesting as the presence of both identical and different *glyA1*/*glyA2 *alleles within an ST), and only one putative lateral transfer event was identified at *glyA*. Therefore, inclusion of *glyA1 *in the typing scheme is merited, since the large number of *glyA *alleles identified in this study would substantially enhance *Arcobacter *strain discrimination.

## Conclusion

The large number of MLST alleles and STs identified in this study indicates that the *Arcobacter *MLST method described here is useful for strain discrimination for the three major *Arcobacter species*, i.e. *A. butzleri*, *A. cryaerophilus *and *A. skirrowii*, as well as two additional *Arcobacter *species, *A. thereius *and *A. cibarius*. Additional genomic sequence data should permit revision and expansion of this typing method into additional *Arcobacter *species. No association, with either host or geographical source, of *Arcobacter *alleles or STs was observed in this study; however, the large suite of alleles and STs present within this sample set make identification of such associations difficult, since most alleles and STs were observed infrequently. Typing of additional *Arcobacter *isolates, thereby increasing potentially the numbers of each allele and ST, may reveal heretofore undetected association patterns within this genus. The increasing association of arcobacters with human illness, transmitted potentially by contaminated food or water, makes this method a valuable addition to *Arcobacter *typing. This method should prove useful in investigations of sporadic and outbreak arcobacterioses and *Arcobacter *epidemiology.

## Methods

### *Arcobacter *strains

The *A. butzleri *set typed in this study consisted of 275 isolates from 16 countries across four continents (N. America, Europe, Asia and Africa), and from a wide variety of food sources and animals (Tables 1 and 2); additionally 102 strains (37%) were isolated from both healthy and diarrheal human stool samples [see additional file 2 - Table S2]. Furthermore, to assess the versatility of the *Arcobacter *MLST method in typing strains of non-*butzleri *species, we assembled a set of isolates from four other *Arcobacter *species: *A. cryaerophilus*, *A. skirrowii*, *A. cibarius *and *A. thereius*. The size and scope of the non-*butzleri *sample set was limited necessarily by the relatively few isolates available for the non-*butzleri *species. Nevertheless, 99 non-*butzleri *isolates were assembled. The majority of these were *A. cryaerophilus *(N = 72) and *A. skirrowii *(N = 15), obtained predominantly from cattle and swine; the remainder included eight *A. cibarius *strains and four *A. thereius *strains. A large number of strains in the *Arcobacter *strain set were of unknown origin (N = 57; 15%).

### Growth conditions and chemicals

All *Arcobacter *strains were cultured routinely under aerobic conditions at 30°C on Brain Heart Infusion agar (Becton Dickinson, Sparks, MD) supplemented with 5% (v/v) laked horse blood (Hema Resource & Supply, Aurora, OR). *Arcobacter halophilus *was grown on Brain Heart Infusion -blood media supplemented with 4% (w/v) NaCl. PCR enzymes and reagents were purchased from New England Biolabs (Beverly, MA) or Epicentre (Madison, WI). All chemicals were purchased from Sigma-Aldrich Chemicals (St. Louis, MO) or Fisher Scientific (Pittsburgh, PA). DNA sequencing chemicals and capillaries were purchased from Applied Biosystems (Foster City, CA). PCR and sequencing oligonucleotides were purchased from MWG-Biotech (High Point, NC).

### Multilocus sequence typing (MLST)

MLST primer sets are listed in Table S1 [see additional file 1]. Each MLST amplification mixture contained: 50 ng genomic DNA, 1 × MasterAmp PCR buffer (Epicentre, Madison, WI), 1 × MasterAmp PCR enhancer (Epicentre), 2.5 mM MgCl_2_, 250 μM (each) dNTPs, 50 pmol each primer, and 1 U *Taq *polymerase (New England Biolabs, Beverly, MA). PCRs for MLST were performed on a Tetrad thermocycler (Bio-Rad, Hercules, CA) with the following settings: 30 cycles of 94°C for 30 sec, 53°C for 30 sec, and 72°C for 2 min. Amplicons were purified on a BioRobot 8000 workstation (Qiagen, Valencia, CA). Cycle sequencing reactions were performed on a Tetrad thermocycler, using the ABI PRISM BigDye terminator cycle sequencing kit (version 3.1; Applied Biosystems, Foster City, CA) and standard protocols. Cycle sequencing extension products were purified using BigDye XTerminator (Applied Biosystems). DNA sequencing was performed on an ABI PRISM 3730 DNA Analyzer (Applied Biosystems), using POP-7 polymer and ABI PRISM Genetic Analyzer Data Collection and ABI PRISM Genetic Analyzer Sequencing Analysis software.

### MLSTparser3 and allele number/sequence type assignment

The Perl program MLSTparser [27] was modified to create the program MLSTparser3. The new features of MLSTparser3 include: 1) incorporation of the MLST schemes for *C. fetus*, *C. insulaenigrae *and the novel *Arcobacter *MLST schemes described in this study, in addition to the original MLST schemes for *C. jejuni*, *C. coli*, *C. lari*, *C. upsaliensis *and *C. helveticus*; 2) automatic association of allele with species, based on phylogenetic analyses of the ten MLST loci present in the different *Campylobacter*/*Arcobacter *MLST methods, permitting immediate identification of chimeras; and 3) automatic assignment of sequence type (ST), based on the profile of seven MLST alleles. Novel alleles and STs are flagged by MLSTparser3 and assigned an arbitrary number.

MLSTparser3 was used to identify the MLST alleles and ST of each *Arcobacter *strain typed in this study. A new *Arcobacter *MLST database was created http://pubmlst.org/arcobacter/; allele and ST data generated in this study were deposited in this database and are available online.

### Phylogenetic analyses

Variable sites and calculation of the *d*_*n*_/*d*_*s *_ratios were performed using START2 http://pubmlst.org/software/analysis/. A dendrogram of unique *Arcobacter *STs was constructed by concatenating the allele sequences comprising each ST. Allele sequences for each strain were concatenated in the order *aspA*-*atpA*-*glnA*-*gltA*-*glyA*-*pgm*-*tkt *for a final composite length of 3341 bp; in addition, the MLST alleles of the *A. halophilus *strain LA31B were extracted from the draft genome (Miller et al., unpublished data), concatenated and incorporated into the ST analysis. Sequence alignments were performed using CLUSTALX (ver. 2.0.5; http://www.clustal.org/), and dendrograms were constructed using the neighbor-joining method with the Kimura 2-parameter distance estimation method. Phylogenetic analyses were performed using MEGA version 4 [36].

## Authors' contributions

WGM designed the research project. WGM designed the MLST primer sets, analyzed the MLST data, performed the phylogenetic analyses and was the principal author of the manuscript. GW and EY isolated the *Arcobacter *genomic DNA, and GW and EY performed the multilocus sequence typing. IVW, SLWO, KH, FM, AS and CJM isolated the *Arcobacter *strains and performed the initial characterization/speciation of the isolates. All authors approved and read the final manuscript.

## Supplementary Material

Additional file 1 **Primers for amplification and sequencing of the seven *Arcobacter *spp. MLST genes**. Primer pairs used for amplifying the MLST loci of *A. butzleri*, *A. cryaerophilus*, *A. skirrowii*, *A. cibarius *and *A. thereius *are listed. For each MLST locus, the allele size is given and for each primer pair the expected amplicon size is provided. Click here for file

Additional file 2 ***Arcobacter *allele numbers and sequence types**. List of allele numbers and sequence types for the 374 arcobacters typed in this study. For each strain, the source and geographic origin is provided (if known). Click here for file
