# Supplementary material for: First multi-locus sequence typing scheme for Arcobacter spp
Source: BMC Microbiol. 2009 Sep 14;9:196. doi: 10.1186/1471-2180-9-196 (PMC2755481; doi:10.1186/1471-2180-9-196)
Supplement: Additional file 1 — Primers for amplification and sequencing of the seven Arcobacter spp. MLST genes. Primer pairs used for amplifying the MLST loci of A. butzleri, A. cryaerophilus, A. skirrowii, A. cibarius and A. thereius are listed. For each MLST locus, the allele size is given and for each primer pair the expected amplicon size is provided. [file 1471-2180-9-196-S1.pdf]

Table S1. Primers for amplification and sequencing of the seven *Arcobacter* spp. MLST genes.

		Oligonucleotide primer set									
	Allele size	Forward (5' → 3')		Reverse (5' → 3')		Amplicon size[a]					
Locus		Primer	Sequence	Primer	Sequence	B	C	S	T	Cb	
aspA	477	aspABF	ATTTTRAGAGATTCTTTTCRCRATAAA	aspABR	AACATTATTCATACAAATTTCAGSATT	**Y**	V	V	V	V	711
		aspACF2	AAATATTRMGAGATGCTTTTTATGGAA	aspACR2	TACAAACTTCAGGATTWGCWGTAAT		**Y**				702
		aspASF	GCTTATCCAACTGCWATTAAAMTTACA	aspASR	CTTCAGGATTTGCTGYAATTCC			**Y**	**Y**		760
		aspACibF	AGCCTTAAGATTTTAAGAGATGCTTTC	aspACibR	AGGATTTGCTGTAATTCCTTTTATACA					**Y**	699
atpA	489	atpABF	CWGTTGCKATTGATACAATTCTTAA	atpABR	CAATTTGTTTTTCAATAACTAATGGTTT	**Y**					774
		atpACF	GATACAATTCTTAAYCAAAAAGGTGA	atpACR	AAAACTTCWACCATTCTTTGWCCAA		**Y**	**Y**	**Y**	**Y**	719
glnA	474	glnABF	TGCAGTTAGTGCWTCTMCTTTAGATAA	glnABR	ATAGRTTTTTCCCATCTTTCCAA	**Y**					734
		glnACF2	AAATGGAATGCCTTTTGATGGTT	glnACR1	TTRTCWCCATAAAGWGGTTTTGGCA		**Y**	**Y**	**Y**	**Y**	657
gltA	429	gltABF	TTGATGGAGARAATTCTGAGTTAAG	gltABR	GGAACATTTTTAACATCACCAATTA	**Y**					706
		gltACF	TGATATWGCTGATTTRGCTGGTAAA	gltACR	CCAATCATTCTTARYTGATCCATAAC		**Y**	**Y**	**Y**	**Y**	651
glyA[b]	507	ABlysS	AAATGGAYGARGAYTTTGTWAATGC	glyABR	CATCTTTYCCTGAAAATGGTTTATT	**Y**					1189
		ABlysS	AAATGGAYGARGAYTTTGTWAATGC	glyACR	GCATCTTTTCCWSWRAATGGTTTAT		**Y**	**Y**	**Y**	**Y**	~1200[c]
		glyABNF	ATGCAGAAGGTTATCCATATAAAAG	ABada2	GTAACACCWACATACTCTTTRAATACTCT	**Y**	V	V	**Y**	**Y**	~1300[c]
		glyACF	TGCAAATGTTCAACCWCATAGTGGA	ABada3	AWAAGWGCYTTCCAWACATTTATTT		**Y**	**Y**			~1300[c]
		glyACF	TGCAAATGTTCAACCWCATAGTGGA	ABada8	TTTTCTTTCWATKCCCCATCT		**Y**	**Y**			~1300[c]
		glyABF	TGGWTGTAAATWTGCAAATGTTCAA	glyABR	CATCTTTYCCTGAAAATGGTTTATT	**Y**					735
		glyACF	TGCAAATGTTCAACCWCATAGTGGA	glyACR	GCATCTTTTCCWSWRAATGGTTTAT		**Y**	**Y**	**Y**	**Y**	723
pgm	503	pgmABF1	TCCRAAAAATYTRACWYTAAAAGGT	pgmABR	AAAGTCTRATTTTATTYTCTGTKCC	**Y**	N	N	N	N	758
		pgmACF1	GGATTAAGAATTGTYCTTGAYTGTGC	pgmACR	TCAACATCTTTTYTATTTTTACCTTCA		**Y**				767
		pgmASF1	AAAAGGKCTTAGAATTGTWCTTGATTG	pgmACR	TCAACATCTTTTYTATTTTTACCTTCA			**Y**	**Y**		771
		pgmACibF	TTCCCAAAAGATTTAACGCTAAAAG	pgmACR	TCAACATCTTTTYTATTTTTACCTTCA					**Y**	791
tkt	462	tktABF	GCTGATATTGCAACAGTWTTAAGTA	tktABR	TAAATCCWGCTTTTTCTTTWGATTT	**Y**	V	V	V	V	721
		tktACF	CTCCTATGGGAMTKGCTGATATTG	tktACR2	ATTAAAYCCAGCTTTTATTTTTGCTTG		**Y**	**Y**	**Y**		737
		tktACF	CTCCTATGGGAMTKGCTGATATTG	tktACibR	GGATTAAATCCTGCTTTTTCTTTAGATT					V	739

Shaded primer pairs represent components of the *A. butzleri*, *A. cryaerophilus*, *A. skirrowii* and *A. thereius* MLST methods.

a. B: *butzleri*; C: *cryaerophilus*; S: *skirrowii*; T: *thereius*; Cb: *cibarius*. V: weak amplification of some strains within a species.

b. ABlys and ABada primer pairs for amplification of *glyA1* or *glyA2*, respectively; glyABF/ABR and glyACF/ACR primer pairs for sequencing only.

c. Size approximate due to regions of variable length directly upstream of *glyA1* and downstream of *glyA2*.
