# Supplementary material for: First multi-locus sequence typing scheme for Arcobacter spp
Source: BMC Microbiol. 2009 Sep 14;9:196. doi: 10.1186/1471-2180-9-196 (PMC2755481; doi:10.1186/1471-2180-9-196)
Supplement: Additional file 2 — Arcobacter allele numbers and sequence types. List of allele numbers and sequence types for the 374 arcobacters typed in this study. For each strain, the source and geographic origin is provided (if known). [file 1471-2180-9-196-S2.pdf]

Table S2. ***Arcobacter*** **allele numbers and sequence types.**

Strain	Other designation	*aspA*	*atpA*	*glnA*	*gltA*	*glyA1*	*glyA2*	*pgm*	*tkt*	ST	Species	Source	Country (State)	Year	Depositor[a]/Reference[b]
RM4018	ATCC 49616^{T}	1	1	1	1	1	142	1	1	ST-1	*butzleri*	Human; stool	US (CA)	1990	
14126	456H-2005	2	2	10	10	10	11	11	10	ST-2	*butzleri*	Human	France	2005	F. Megraud
R-325		2	2	10	10	10	11	11	10	ST-2	*butzleri*	Human	Belgium		K. Houf
14179	STF-36	2	2	24	27	112	112	35	20	ST-3	*butzleri*	Fresh pork	Thailand	2003	C. Mason, AFRIMS
14185	H-02	2	2	1	15	45	11	40	10	ST-4	*butzleri*	Healthy child's stool	Vietnam	2001	C. Mason, AFRIMS
14186	H-03	2	2	1	15	45	11	40	10	ST-4	*butzleri*	Healthy child's stool	Vietnam	2001	C. Mason, AFRIMS
14187	H-04	2	2	1	15	45	11	40	10	ST-4	*butzleri*	Healthy child's stool	Vietnam	2001	C. Mason, AFRIMS
14193	H-10	2	2	1	15	45	11	40	10	ST-4	*butzleri*	Child's diarrheal stool	Vietnam	2001	C. Mason, AFRIMS
RM4467	NADC 3566	2	2	2	2	11	11	2	2	ST-5	*butzleri*	Primate	US		I. Wesley, NADC
14107	56-2004	2	2	2	2	11	11	2	2	ST-5	*butzleri*	Human; stool	France	2004	F. Megraud
14233	LPF-17	2	23	24	19	75	75	60	46	ST-6	*butzleri*	Fresh chicken	Thailand	2003	C. Mason, AFRIMS
RM5529	CDC D1751	2	59	45	15	151	152	113	2	ST-7	*butzleri*	Human; stool	US (TX)	**(c)**	Kiehlbauch et al, 1991
RM5538	CDC D2725	3	2	20	30	160	160	107	82	ST-8	*butzleri*	Human; stool	US (MA)	**(c)**	Kiehlbauch et al, 1991
14151	STF-08	3	3	3	2	17	18	17	3	ST-9	*butzleri*	Fresh beef	Thailand	2003	C. Mason, AFRIMS
14109	78-2004	3	3	3	3	2	2	3	3	ST-10	*butzleri*	Human; stool	France	2004	F. Megraud
14121	1285-2003	3	6	6	6	6	6	6	5	ST-11	*butzleri*	Human; stool	France	2003	F. Megraud
14159	STF-16	3	17	16	20	25	25	21	7	ST-12	*butzleri*	Fresh chicken	Thailand	2003	C. Mason, AFRIMS
14224	LPF-08	3	17	17	20	67	67	53	44	ST-13	*butzleri*	Fresh chicken	Thailand	2003	C. Mason, AFRIMS
RM5549	CDC D2784	3	24	2	45	166	166	120	84	ST-14	*butzleri*	Human; stool	Thailand	**(c)**	Kiehlbauch et al, 1991
RM5550	CDC D2786	3	24	2	45	166	166	120	84	ST-14	*butzleri*	Human; stool	Thailand	**(c)**	Kiehlbauch et al, 1991
RM5554	CDC D2791	3	32	3	34	168	46	44	36	ST-15	*butzleri*	Human; stool	Thailand	**(c)**	Kiehlbauch et al, 1991
14194	H-11	3	32	3	34	168	46	44	36	ST-15	*butzleri*	Child's diarrheal stool	Vietnam	2002	C. Mason, AFRIMS
RM5230	RIGS 15342	3	55	30	15	136	136	104	36	ST-16	*butzleri*	Human; stool	Denmark	1997	S. On
14112	265H-2004	4	4	4	4	3	3	4	89	ST-17	*butzleri*	Human; stool	France	2004	F. Megraud
14116	559H-2003	4	4	4	4	3	3	4	89	ST-17	*butzleri*	Human; stool	France	2003	F. Megraud
RM5235	DVI TU25	4	4	4	4	139	216	4	89	ST-18	*butzleri*	Turkey	Denmark	2001	On et al., 2004
AB6		4	39	51	64	184	185	125	87	ST-19	*butzleri*	Bovine	Belgium		K. Houf
AB7		4	39	51	64	184	185	125	87	ST-19	*butzleri*	Bovine	Belgium		K. Houf
RM5528	CDC D1106	4	58	44	13	190	150	4	89	ST-20	*butzleri*	Human; stool	US (MT)	**(c)**	Kiehlbauch et al, 1991
RM4844	NADC 5691	5	5	5	19	121	121	86	10	ST-21	*butzleri*	Ground pork	US		I. Wesley, NADC
RM4845	NADC 5692	5	5	5	19	121	121	86	10	ST-21	*butzleri*	Ground pork	US		I. Wesley, NADC
14144	STF-01	5	5	12	12	83	83	13	12	ST-22	*butzleri*	Fresh beef	Thailand	2003	C. Mason, AFRIMS
RM5566	NADC 4496; 13(8/18)	5	5	5	5	179	5	11	4	ST-23	*butzleri*	Swine	Unknown		I. Wesley, NADC
RM5592	NADC 4649; 138	5	5	9	5	8	5	50	85	ST-24	*butzleri*	Swine	Unknown		I. Wesley, NADC
RM5593	NADC 4652; 155	5	5	9	5	8	5	50	85	ST-24	*butzleri*	Swine	Unknown		I. Wesley, NADC
RM5595	NADC 4691; 252	5	5	9	5	8	5	50	85	ST-24	*butzleri*	Swine	Unknown		I. Wesley, NADC
14175	STF-32	5	5	1	12	83	83	33	30	ST-25	*butzleri*	Fresh pork	Thailand	2003	C. Mason, AFRIMS
14118	1172-2003	5	5	5	5	4	5	5	4	ST-26	*butzleri*	Human; stool	France	2003	F. Megraud
14119	1197-2003	5	5	5	5	4	5	5	4	ST-26	*butzleri*	Human; stool	France	2003	F. Megraud
14120	1188-2003	5	5	5	5	4	5	5	4	ST-26	*butzleri*	Human; stool	France	2003	F. Megraud
14227	LPF-11	5	5	24	37	68	69	2	20	ST-27	*butzleri*	Fresh chicken	Thailand	2003	C. Mason, AFRIMS
14240	LPF-25	5	5	24	27	79	80	62	20	ST-28	*butzleri*	Fresh chicken	Thailand	2003	C. Mason, AFRIMS
14241	LPF-26	5	5	24	27	79	80	62	20	ST-28	*butzleri*	Fresh chicken	Thailand	2003	C. Mason, AFRIMS
14246	LPF-31	5	5	1	12	83	83	64	12	ST-29	*butzleri*	Fresh pork	Thailand	2003	C. Mason, AFRIMS
14167	STF-24	5	12	7	9	33	66	7	24	ST-30	*butzleri*	Fresh chicken	Thailand	2003	C. Mason, AFRIMS
14172	STF-29	5	12	11	26	36	66	30	24	ST-31	*butzleri*	Fresh chicken	Thailand	2003	C. Mason, AFRIMS
14217	LPF-01	5	12	31	26	66	66	7	43	ST-32	*butzleri*	Fresh pork	Thailand	2003	C. Mason, AFRIMS

Strain	Other designation	*aspA*	*atpA*	*glnA*	*gltA*	*glyA1*	*glyA2*	*pgm*	*tkt*	ST	Species	Source	Country (State)	Year	Depositor[a]/Reference[b]
14218	LPF-02	5	12	11	26	60	66	30	24	ST-33	*butzleri*	Fresh pork	Thailand	2003	C. Mason, AFRIMS
14223	LPF-07	5	12	7	26	66	66	7	24	ST-34	*butzleri*	Fresh chicken	Thailand	2003	C. Mason, AFRIMS
14225	LPF-09	5	12	7	26	66	66	7	24	ST-34	*butzleri*	Fresh chicken	Thailand	2003	C. Mason, AFRIMS
14231	LPF-15	6	2	2	19	73	73	58	20	ST-35	*butzleri*	Fresh pork sausage	Thailand	2003	C. Mason, AFRIMS
RM4849	NADC 5699	6	5	38	7	144	144	93	13	ST-36	*butzleri*	Ground pork	US		I. Wesley, NADC
14122	1340-2003	6	5	7	7	7	8	7	6	ST-37	*butzleri*	Human; stool	France	2003	F. Megraud
14200	H-17	6	12	1	12	49	50	49	39	ST-38	*butzleri*	Child's diarrheal stool	Thailand	2005	C. Mason, AFRIMS
14203	H-20	6	12	1	12	49	50	49	39	ST-38	*butzleri*	Healthy child's stool	Thailand	2005	C. Mason, AFRIMS
RM4840	NADC 5274	6	20	20	17	118	118	91	23	ST-39	*butzleri*	Chicken carcass	US		I. Wesley, NADC
RM5212	Lagos 12	6	20	20	17	72	72	26	23	ST-40	*butzleri*	Poultry abattoir effluent	Nigeria	2000	S. On
14165	STF-22	6	20	20	17	72	72	26	23	ST-40	*butzleri*	Fresh chicken	Thailand	2003	C. Mason, AFRIMS
14176	STF-33	6	20	20	17	72	38	26	24	ST-41	*butzleri*	Fresh pork	Thailand	2003	C. Mason, AFRIMS
14230	LPF-14	6	20	20	41	72	72	26	23	ST-42	*butzleri*	Fresh chicken	Thailand	2003	C. Mason, AFRIMS
14177	STF-34	6	26	23	19	39	98	34	31	ST-43	*butzleri*	Fresh pork	Thailand	2003	C. Mason, AFRIMS
14236	LPF-21	6	26	23	19	98	98	34	31	ST-44	*butzleri*	Fresh chicken	Thailand	2003	C. Mason, AFRIMS
14202	H-19	6	34	1	12	120	120	50	14	ST-45	*butzleri*	Healthy child's stool	Thailand	2005	C. Mason, AFRIMS
14123	1407-2003	7	7	7	7	186	186	8	7	ST-46	*butzleri*	Human; stool	France	2003	F. Megraud
14124	1426-2003	8	8	8	8	9	191	9	8	ST-47	*butzleri*	Human; stool	France	2003	F. Megraud
14125	1477-2003	9	5	9	9	120	120	10	9	ST-48	*butzleri*	Human; stool	France	2003	F. Megraud
14128	CIP 103727	10	9	11	11	12	192	12	11	ST-49	*butzleri*	Human	France		F. Megraud
RM5564	NADC 4485; 64(7/6)f	10	20	11	19	177	177	123	11	ST-50	*butzleri*	Swine	Unknown		I. Wesley, NADC
14239	LPF-24	10	39	1	19	78	78	61	29	ST-51	*butzleri*	Fresh chicken	Thailand	2003	C. Mason, AFRIMS
RM4591	NADC 5256	11	2	11	44	56	56	84	40	ST-52	*butzleri*	Turkey	US		I. Wesley, NADC
RM4463	NADC 3554	11	4	1	49	106	106	78	52	ST-53	*butzleri*	Human	US (CO)		I. Wesley, NADC
14145	STF-02	11	10	11	13	13	13	14	13	ST-54	*butzleri*	Fresh beef	Thailand	2003	C. Mason, AFRIMS
14228	LPF-12	11	12	11	19	70	70	24	11	ST-55	*butzleri*	Fresh chicken	Thailand	2003	C. Mason, AFRIMS
14146	STF-03	12	11	2	14	14	61	8	14	ST-56	*butzleri*	Fresh beef	Thailand	2003	C. Mason, AFRIMS
14150	STF-07	12	11	2	14	14	61	8	14	ST-56	*butzleri*	Fresh beef	Thailand	2003	C. Mason, AFRIMS
14153	STF-10	12	11	2	14	14	61	8	14	ST-56	*butzleri*	Fresh beef	Thailand	2003	C. Mason, AFRIMS
14220	LPF-04	12	11	2	14	14	61	8	14	ST-56	*butzleri*	Fresh pork	Thailand	2003	C. Mason, AFRIMS
14149	STF-06	12	11	2	9	61	61	16	16	ST-57	*butzleri*	Fresh beef	Thailand	2003	C. Mason, AFRIMS
14147	STF-04	13	12	13	15	15	36	8	6	ST-58	*butzleri*	Fresh beef	Thailand	2003	C. Mason, AFRIMS
14154	STF-11	13	12	13	15	15	36	8	6	ST-58	*butzleri*	Fresh beef	Thailand	2003	C. Mason, AFRIMS
14148	STF-05	14	13	14	16	16	16	15	15	ST-59	*butzleri*	Fresh beef	Thailand	2003	C. Mason, AFRIMS
RM4473	NADC 3572	14	17	35	51	111	111	82	57	ST-60	*butzleri*	Human	US (CA)		I. Wesley, NADC
14180	STF-37	14	27	15	28	40	40	36	32	ST-61	*butzleri*	Fresh pork	Thailand	2003	C. Mason, AFRIMS
RM4848	NADC 5698	14	49	26	55	123	123	92	63	ST-62	*butzleri*	Ground pork	US		I. Wesley, NADC
RM5132		14	50	30	25	152	208	95	64	ST-63	*butzleri*	Chicken; drumstick	US (CA)	2005	W. Miller, USDA
RM5535	CDC D2576	14	63	26	62	156	156	118	79	ST-64	*butzleri*	Human; peritoneal fluid	US (CA)	**(c)**	Kiehlbauch et al, 1991
RM5562	CDC D2280	14	68	50	40	174	174	122	79	ST-65	*butzleri*	Human; stool	US (TN)	**(c)**	Kiehlbauch et al, 1991
14152	STF-09	15	10	1	17	19	20	2	13	ST-66	*butzleri*	Fresh beef	Thailand	2003	C. Mason, AFRIMS
14155	STF-12	16	14	1	18	147	22	18	12	ST-67	*butzleri*	Fresh beef	Thailand	2003	C. Mason, AFRIMS
14183	STF-40	16	14	1	18	43	43	39	12	ST-68	*butzleri*	Chicken eggs	Thailand	2003	C. Mason, AFRIMS
14156	STF-13	17	15	15	12	23	23	19	17	ST-69	*butzleri*	Fresh beef	Thailand	2003	C. Mason, AFRIMS
14157	STF-14	17	15	15	12	23	23	19	17	ST-69	*butzleri*	Fresh beef	Thailand	2003	C. Mason, AFRIMS
RM5533	CDC D2563	17	62	46	55	24	24	116	78	ST-70	*butzleri*	Human; blood	US (MA)	**(c)**	Kiehlbauch et al, 1991
RM5555	CDC D2828	17	62	26	53	169	169	121	79	ST-71	*butzleri*	Human; stool	Canada	**(c)**	Kiehlbauch et al, 1991
14158	STF-15	18	16	11	19	24	24	20	18	ST-72	*butzleri*	Fresh chicken	Thailand	2003	C. Mason, AFRIMS

Strain	Other designation	*aspA*	*atpA*	*glnA*	*gltA*	*glyA1*	*glyA2*	*pgm*	*tkt*	ST	Species	Source	Country (State)	Year	Depositor[a]/Reference[b]
14164	STF-21	18	16	11	19	24	24	20	18	ST-72	*butzleri*	Fresh chicken	Thailand	2003	C. Mason, AFRIMS
RM5214	Lagos 18	19	17	17	12	67	210	77	65	ST-73	*butzleri*	Poultry abattoir effluent	Nigeria	2000	On et al., 2004
14160	STF-17	19	17	17	20	26	27	22	19	ST-74	*butzleri*	Fresh chicken	Thailand	2003	C. Mason, AFRIMS
RM5521	CDC D2810	20	2	2	30	143	143	110	71	ST-75	*butzleri*	Human; stool	US (OH)	**(c)**	Kiehlbauch et al, 1991
14213	H-30	20	2	11	22	56	56	55	40	ST-76	*butzleri*	Healthy adult's stool	Thailand	2001	C. Mason, AFRIMS
14257	LPF-42	20	7	25	19	93	93	71	14	ST-77	*butzleri*	Fresh chicken	Thailand	2003	C. Mason, AFRIMS
LMG14714		20	7	20	15	186	186	8	14	ST-78	*butzleri*	Unknown	Greece		
14181	STF-38	20	7	25	17	41	41	37	33	ST-79	*butzleri*	Fresh pork	Thailand	2003	C. Mason, AFRIMS
14251	LPF-36	20	7	20	23	186	186	45	37	ST-80	*butzleri*	Fresh river fish	Thailand	2003	C. Mason, AFRIMS
R-8636		20	12	11	19	189	221	127	88	ST-81	*butzleri*	Human	Belgium		K. Houf
14161	STF-18	20	18	18	21	28	29	23	20	ST-82	*butzleri*	Fresh chicken	Thailand	2003	C. Mason, AFRIMS
14195	H-12	20	23	20	23	186	186	45	37	ST-83	*butzleri*	Child's diarrheal stool	Vietnam	2002	C. Mason, AFRIMS
RM1591	NADC 5377	20	25	7	2	102	196	32	2	ST-84	*butzleri*	Turkey	US		I. Wesley, NADC
14174	STF-31	20	25	7	2	37	196	32	2	ST-85	*butzleri*	Fresh chicken	Thailand	2003	C. Mason, AFRIMS
RM4470	NADC 3569	20	27	36	48	109	110	81	55	ST-86	*butzleri*	Primate	US		I. Wesley, NADC
RM4127	SSI 71032	20	39	34	19	104	201	76	51	ST-87	*butzleri*	Human	Denmark		S. On
RM4471	NADC 3570	20	39	4	11	110	110	63	56	ST-88	*butzleri*	Primate	US		I. Wesley, NADC
RM4610	NADC 3563	20	39	7	11	187	187	87	55	ST-89	*butzleri*	Human	US		I. Wesley, NADC
RM5226	Kafkas 22	20	39	19	15	133	187	87	58	ST-90	*butzleri*	Chicken carcass	Turkey	2000	On et al., 2004
R-14600		20	39	19	15	133	187	87	58	ST-90	*butzleri*	Chicken carcass	Turkey		K. Houf
R-14606		20	39	19	15	133	187	87	58	ST-90	*butzleri*	Chicken carcass	Turkey		K. Houf
RM5227	Kafkas 41	20	39	40	19	134	117	102	2	ST-91	*butzleri*	Chicken carcass	Turkey	2000	On et al., 2004
RM5238	BU13F	20	57	42	30	140	140	107	14	ST-92	*butzleri*	Poultry feces	UK	1996	S. On
14162	STF-19	21	19	19	22	30	30	24	21	ST-93	*butzleri*	Fresh chicken	Thailand	2003	C. Mason, AFRIMS
14168	STF-25	21	22	21	24	48	48	27	25	ST-94	*butzleri*	Fresh chicken	Thailand	2003	C. Mason, AFRIMS
14199	H-16	21	22	21	24	48	48	27	25	ST-94	*butzleri*	Child's diarrheal stool	Thailand	2004	C. Mason, AFRIMS
14259	LPF-44	21	22	21	24	48	48	27	25	ST-94	*butzleri*	Fresh pork	Thailand	2003	C. Mason, AFRIMS
14163	STF-20	22	7	1	14	31	31	25	22	ST-95	*butzleri*	Fresh chicken	Thailand	2003	C. Mason, AFRIMS
RM4479	NADC 9563	23	5	24	2	124	199	35	20	ST-96	*butzleri*	Turkey carcass	US		I. Wesley, NADC
RM4482	NADC 9566	23	5	24	2	124	199	35	20	ST-96	*butzleri*	Turkey carcass	US		I. Wesley, NADC
RM5224	Kafkas 7	23	5	24	44	80	112	35	55	ST-97	*butzleri*	Chicken carcass	Turkey	2000	On et al., 2004
14242	LPF-27	23	5	24	2	81	199	63	20	ST-98	*butzleri*	Fresh chicken	Thailand	2003	C. Mason, AFRIMS
14254	LPF-39	23	7	1	15	89	90	69	18	ST-99	*butzleri*	Fresh chicken	Thailand	2003	C. Mason, AFRIMS
RM4839	NADC 5273	23	7	11	44	5	5	90	62	ST-100	*butzleri*	Chicken carcass	US		I. Wesley, NADC
RM5225	Kafkas 10	23	7	41	19	132	132	101	55	ST-101	*butzleri*	Poultry	Turkey		On et al., 2004
RM5563	NADC 3733; 12934 Kid A lg	23	7	40	15	175	176	26	58	ST-102	*butzleri*	Swine	Unknown		I. Wesley, NADC
RM5565	NADC 4486; 80(7/6)	23	7	7	15	178	219	40	58	ST-103	*butzleri*	Swine	Unknown		I. Wesley, NADC
RM5579	NADC 3453; 92-6570	23	7	40	15	181	98	102	58	ST-104	*butzleri*	Unknown	Unknown		I. Wesley, NADC
RM5218	Lagos 34	23	41	33	13	129	35	70	31	ST-105	*butzleri*	Poultry abattoir effluent	Nigeria	2000	On et al., 2004
14255	LPF-40	23	41	33	13	129	91	70	31	ST-105	*butzleri*	Fresh chicken	Thailand	2003	C. Mason, AFRIMS
RM4480	NADC 9564	23	44	24	2	170	112	62	20	ST-106	*butzleri*	Turkey carcass	US		I. Wesley, NADC
RM4481	NADC 9565	23	44	24	2	170	112	62	20	ST-106	*butzleri*	Turkey carcass	US		I. Wesley, NADC
RM4484	NADC 9568	23	44	24	2	170	112	62	20	ST-106	*butzleri*	Turkey carcass	US		I. Wesley, NADC
RM5231	DVI DU12	23	44	24	2	137	112	62	20	ST-107	*butzleri*	Duck	Denmark	2001	On et al., 2004
RM5233	DVI 21F	23	44	24	15	124	124	55	20	ST-108	*butzleri*	Chicken	Denmark	2001	On et al., 2004
RM5236	BU 21C1	23	44	24	15	124	124	55	20	ST-108	*butzleri*	Poultry	UK	1995	S. On
14169	STF-26	24	23	22	25	34	194	28	26	ST-109	*butzleri*	Fresh chicken	Thailand	2003	C. Mason, AFRIMS
14170	STF-27	25	3	1	7	35	195	29	27	ST-110	*butzleri*	Fresh chicken	Thailand	2003	C. Mason, AFRIMS

Strain	Other designation	*aspA*	*atpA*	*glnA*	*gltA*	*glyA1*	*glyA2*	*pgm*	*tkt*	ST	Species	Source	Country (State)	Year	Depositor[a]/Reference[b]
14197	H-14	25	31	26	35	52	52	47	36	ST-111	*butzleri*	Child's diarrheal stool	Vietnam	2002	C. Mason, AFRIMS
14208	H-25	25	31	26	35	52	52	47	36	ST-111	*butzleri*	Healthy child's stool	Thailand	2005	C. Mason, AFRIMS
RM5530	CDC D1780	26	11	19	12	153	153	114	75	ST-112	*butzleri*	Human; abdominal contents	US (MA)	**(c)**	Kiehlbauch et al, 1991
14171	STF-28	26	24	17	20	67	67	22	28	ST-113	*butzleri*	Fresh chicken	Thailand	2003	C. Mason, AFRIMS
14256	LPF-41	27	25	7	2	102	92	31	29	ST-114	*butzleri*	Fresh chicken	Thailand	2003	C. Mason, AFRIMS
14173	STF-30	27	25	7	2	15	92	31	29	ST-115	*butzleri*	Fresh chicken	Thailand	2003	C. Mason, AFRIMS
14178	STF-35	28	5	7	7	167	167	7	6	ST-116	*butzleri*	Fresh pork	Thailand	2003	C. Mason, AFRIMS
RM5543	CDC D2776	28	5	7	7	120	167	7	6	ST-117	*butzleri*	Human; stool	Thailand	**(c)**	Kiehlbauch et al, 1991
RM5551	CDC D2787	28	5	7	7	120	167	7	6	ST-117	*butzleri*	Human; stool	Thailand	**(c)**	Kiehlbauch et al, 1991
14189	H-06	28	5	7	7	120	167	7	6	ST-117	*butzleri*	Healthy child's stool	Vietnam	2002	C. Mason, AFRIMS
14207	H-24	28	5	7	7	120	167	7	6	ST-117	*butzleri*	Child's diarrheal stool	Thailand	2004	C. Mason, AFRIMS
14219	LPF-03	28	5	7	7	120	167	7	6	ST-117	*butzleri*	Fresh pork	Thailand	2003	C. Mason, AFRIMS
14182	STF-39	29	28	26	29	42	197	38	34	ST-118	*butzleri*	Fresh pork	Thailand	2003	C. Mason, AFRIMS
RM4464	NADC 3556	30	5	5	30	120	120	35	4	ST-119	*butzleri*	Human	US (KS)		I. Wesley, NADC
RM4485	NADC 9569	30	5	5	30	120	120	83	58	ST-120	*butzleri*	Turkey carcass	US		I. Wesley, NADC
RM5124		30	5	5	30	125	120	35	4	ST-121	*butzleri*	Chicken; thighs	US (CA)	2005	W. Miller, USDA
RM5126		30	5	5	30	36	120	35	4	ST-122	*butzleri*	Chicken; breast	US (CA)	2005	W. Miller, USDA
RM5133		30	5	5	30	36	120	35	4	ST-122	*butzleri*	Chicken; breast	US (CA)	2005	W. Miller, USDA
RM5567	NADC 4498; 18(8/18)f	30	5	9	19	180	120	101	55	ST-123	*butzleri*	Swine	Unknown		I. Wesley, NADC
RM5568	NADC 4613; 59	30	5	9	19	180	120	101	55	ST-123	*butzleri*	Swine	Unknown		I. Wesley, NADC
RM5591	NADC 4623	30	5	9	19	180	120	101	55	ST-123	*butzleri*	Swine	US (IA)		I. Wesley, NADC
RM5596	NADC 4712; 219	30	5	9	19	180	120	101	55	ST-123	*butzleri*	Swine	Unknown		I. Wesley, NADC
14184	H-01	30	5	5	30	44	120	35	4	ST-124	*butzleri*	Healthy child's stool	Vietnam	2001	C. Mason, AFRIMS
14188	H-05	30	5	5	30	44	120	35	4	ST-124	*butzleri*	Healthy child's stool	Vietnam	2001	C. Mason, AFRIMS
RM5545	CDC D2779	31	29	2	31	65	65	41	35	ST-125	*butzleri*	Human; stool	Thailand	**(c)**	Kiehlbauch et al, 1991
RM5597	NADC 5258; a11 1/30	31	29	2	31	65	65	41	35	ST-125	*butzleri*	Poultry	Unknown		I. Wesley, NADC
14190	H-07	31	29	2	31	65	65	41	35	ST-125	*butzleri*	Healthy child's stool	Vietnam	2002	C. Mason, AFRIMS
RM4465	NADC 3562	31	43	35	15	107	202	79	53	ST-126	*butzleri*	Human	US (CA)		I. Wesley, NADC
RM4468	NADC 3567	31	43	35	50	108	108	80	54	ST-127	*butzleri*	Human	US (CA)		I. Wesley, NADC
RM5520	CDC D2778	32	30	11	32	161	161	42	20	ST-128	*butzleri*	Human; stool	Thailand	**(c)**	Kiehlbauch et al, 1991
RM5539	CDC D2775	32	30	11	32	161	161	42	20	ST-128	*butzleri*	Human; stool	Thailand	**(c)**	Kiehlbauch et al, 1991
RM5553	CDC D2790	32	30	11	32	161	161	42	20	ST-128	*butzleri*	Human; stool	Thailand	**(c)**	Kiehlbauch et al, 1991
14191	H-08	32	30	11	32	161	161	42	20	ST-128	*butzleri*	Healthy child's stool	Vietnam	2002	C. Mason, AFRIMS
RM5546	CDC D2780	33	31	27	33	22	22	43	4	ST-129	*butzleri*	Human; stool	Thailand	**(c)**	Kiehlbauch et al, 1991
RM5547	CDC D2782	33	31	27	33	22	22	43	4	ST-129	*butzleri*	Human; stool	Thailand	**(c)**	Kiehlbauch et al, 1991
RM5552	CDC D2788	33	31	27	33	22	22	43	4	ST-129	*butzleri*	Human; stool	Thailand	**(c)**	Kiehlbauch et al, 1991
14192	H-09	33	31	27	33	22	22	43	4	ST-129	*butzleri*	Healthy child's stool	Vietnam	2002	C. Mason, AFRIMS
14196	H-13	34	12	2	34	58	58	46	38	ST-130	*butzleri*	Child's diarrheal stool	Vietnam	2002	C. Mason, AFRIMS
14215	H-32	34	12	2	34	58	58	46	38	ST-130	*butzleri*	Healthy child's stool	Thailand	2002	C. Mason, AFRIMS
14216	H-33	35	11	2	35	59	59	47	36	ST-131	*butzleri*	Child's diarrheal stool	Thailand	2003	C. Mason, AFRIMS
RM4843	NADC 5278	35	33	28	36	120	207	48	38	ST-132	*butzleri*	Chicken carcass	US		I. Wesley, NADC
RM5556	CDC D2829	35	33	28	36	170	218	48	38	ST-133	*butzleri*	Human; stool	Canada	**(c)**	Kiehlbauch et al, 1991
14198	H-15	35	33	28	36	47	47	48	38	ST-134	*butzleri*	Child's diarrheal stool	Vietnam	2002	C. Mason, AFRIMS
14221	LPF-05	35	33	19	20	62	63	48	38	ST-135	*butzleri*	Fresh chicken	Thailand	2003	C. Mason, AFRIMS
14226	LPF-10	35	33	19	20	62	63	48	38	ST-135	*butzleri*	Fresh chicken	Thailand	2003	C. Mason, AFRIMS
14234	LPF-19	35	33	28	37	76	198	48	38	ST-136	*butzleri*	Fresh chicken	Thailand	2003	C. Mason, AFRIMS
14201	H-18	36	3	29	37	87	87	22	4	ST-137	*butzleri*	Child's diarrheal stool	Thailand	2005	C. Mason, AFRIMS
14204	H-21	36	3	29	37	87	87	22	4	ST-137	*butzleri*	Healthy child's stool	Thailand	2005	C. Mason, AFRIMS

Strain	Other designation	*aspA*	*atpA*	*glnA*	*gltA*	*glyA1*	*glyA2*	*pgm*	*tkt*	ST	Species	Source	Country (State)	Year	Depositor[a]/Reference[b]
RM5523	NADC 3297; 4562	37	5	11	30	144	145	16	72	ST-138	*butzleri*	Human	Unknown		I. Wesley, NADC
RM4129	167.97	37	5	11	17	127	127	16	40	ST-139	*butzleri*	Human	S. Africa	1997	A. Lastovica
RM5213	Lagos 16	37	5	11	17	127	127	16	40	ST-139	*butzleri*	Poultry abattoir effluent	Nigeria	2000	S. On
14205	H-22	37	5	11	17	127	127	16	40	ST-139	*butzleri*	Healthy child's stool	Thailand	2006	C. Mason, AFRIMS
RM3790	29.97	37	7	1	23	103	103	75	33	ST-140	*butzleri*	Human	S. Africa	1997	A. Lastovica
14248	LPF-33	37	23	26	12	187	187	65	10	ST-141	*butzleri*	Fresh squid	Thailand	2003	C. Mason, AFRIMS
14229	LPF-13	37	37	32	40	71	71	57	32	ST-142	*butzleri*	Fresh chicken	Thailand	2003	C. Mason, AFRIMS
RM5540	CDC D2907	38	3	24	63	162	162	59	45	ST-143	*butzleri*	Human; stool	US (MA)	**(c)**	Kiehlbauch et al, 1991
14206	H-23	38	35	26	7	51	51	51	4	ST-144	*butzleri*	Child's diarrheal stool	Thailand	2005	C. Mason, AFRIMS
RM5519	CDC D2720	39	5	1	34	107	107	109	38	ST-145	*butzleri*	Human; stool	US (IN)	**(c)**	Kiehlbauch et al, 1991
RM5542	CDC D2914	39	5	2	39	164	164	109	38	ST-146	*butzleri*	Human; stool	US (IA)	**(c)**	Kiehlbauch et al, 1991
14209	H-26	39	7	17	2	53	53	52	41	ST-147	*butzleri*	Adult diarrheal stool	Thailand	2002	C. Mason, AFRIMS
14214	H-31	39	33	2	39	57	57	56	38	ST-148	*butzleri*	Healthy adult's stool	Thailand	2001	C. Mason, AFRIMS
14262	LPF-47	39	41	30	46	99	99	72	40	ST-149	*butzleri*	Fresh river fish	Thailand	2003	C. Mason, AFRIMS
14263	LPF-48	39	42	27	47	100	200	73	20	ST-150	*butzleri*	Fresh squid	Thailand	2003	C. Mason, AFRIMS
14210	H-27	40	36	3	38	54	54	53	38	ST-151	*butzleri*	Adult diarrheal stool	Thailand	2001	C. Mason, AFRIMS
14211	H-28	41	11	30	12	55	55	54	42	ST-152	*butzleri*	Adult diarrheal stool	Thailand	2001	C. Mason, AFRIMS
14212	H-29	41	11	30	12	55	55	54	42	ST-152	*butzleri*	Adult diarrheal stool	Thailand	2002	C. Mason, AFRIMS
RM4593	NADC 5259	42	25	7	52	65	65	86	14	ST-153	*butzleri*	Turkey	US		I. Wesley, NADC
14222	LPF-06	42	25	7	26	64	65	26	2	ST-154	*butzleri*	Fresh chicken	Thailand	2003	C. Mason, AFRIMS
14232	LPF-16	43	3	2	42	74	74	59	45	ST-155	*butzleri*	Fresh squid	Thailand	2003	C. Mason, AFRIMS
14235	LPF-20	44	38	1	43	144	77	37	33	ST-156	*butzleri*	Fresh chicken	Thailand	2003	C. Mason, AFRIMS
14250	LPF-35	44	38	1	12	88	88	67	33	ST-157	*butzleri*	Fresh river fish	Thailand	2003	C. Mason, AFRIMS
14260	LPF-45	45	25	7	26	96	97	26	2	ST-158	*butzleri*	Fresh pork	Thailand	2003	C. Mason, AFRIMS
14238	LPF-23	45	25	7	26	98	97	26	14	ST-159	*butzleri*	Fresh chicken	Thailand	2003	C. Mason, AFRIMS
14252	LPF-37	45	25	7	26	96	97	26	14	ST-160	*butzleri*	Fresh chicken	Thailand	2003	C. Mason, AFRIMS
14247	LPF-32	46	2	19	6	25	86	24	11	ST-161	*butzleri*	Fresh pork	Thailand	2003	C. Mason, AFRIMS
14243	LPF-28	46	19	19	6	25	82	24	11	ST-162	*butzleri*	Fresh chicken	Thailand	2003	C. Mason, AFRIMS
14244	LPF-29	47	7	2	44	83	83	37	47	ST-163	*butzleri*	Fresh pork	Thailand	2003	C. Mason, AFRIMS
14245	LPF-30	48	25	1	19	84	85	8	6	ST-164	*butzleri*	Fresh pork	Thailand	2003	C. Mason, AFRIMS
14249	LPF-34	49	3	29	37	87	87	66	4	ST-165	*butzleri*	Fresh shrimp	Thailand	2003	C. Mason, AFRIMS
14253	LPF-38	50	40	19	45	165	165	68	48	ST-166	*butzleri*	Fresh chicken	Thailand	2003	C. Mason, AFRIMS
RM5548	CDC D2783	50	40	19	12	173	165	68	48	ST-167	*butzleri*	Human; stool	Thailand	**(c)**	Kiehlbauch et al, 1991
RM5561	CDC D2785	50	40	19	12	173	165	68	48	ST-167	*butzleri*	Human; stool	Thailand	**(c)**	Kiehlbauch et al, 1991
14258	LPF-43	51	16	1	15	94	95	26	49	ST-168	*butzleri*	Fresh pork	Thailand	2003	C. Mason, AFRIMS
14261	LPF-46	52	39	11	19	98	98	58	9	ST-169	*butzleri*	Fresh pork	Thailand	2003	C. Mason, AFRIMS
RM1588	NADC 5276	53	15	15	48	101	101	74	50	ST-170	*butzleri*	Chicken	US		I. Wesley, NADC
RM4462	NADC 3553	54	14	33	15	105	105	77	4	ST-171	*butzleri*	Human	US (TX)		I. Wesley, NADC
RM4592	NADC 5257	55	45	26	48	113	113	85	59	ST-172	*butzleri*	Turkey	US		I. Wesley, NADC
RM5531	CDC D2197	55	60	26	40	154	154	79	76	ST-173	*butzleri*	Human; stool	US (ID)	**(c)**	Kiehlbauch et al, 1991
RM5537	CDC D2638	55	65	48	55	158	159	38	81	ST-174	*butzleri*	Human; stool	US (AZ)	**(c)**	Kiehlbauch et al, 1991
RM4594	NADC 5260	56	2	11	23	114	204	37	20	ST-175	*butzleri*	Turkey	US		I. Wesley, NADC
RM4596	NADC 5262	57	46	37	22	115	46	16	31	ST-176	*butzleri*	Turkey	US		I. Wesley, NADC
RM4597	NADC 5264	58	41	1	15	116	183	70	6	ST-177	*butzleri*	Turkey	US		I. Wesley, NADC
RM4835	NADC 5268	59	47	26	53	117	205	88	60	ST-178	*butzleri*	Turkey	US		I. Wesley, NADC
RM4837	NADC 5271	60	15	15	54	183	183	89	61	ST-179	*butzleri*	Turkey	US		I. Wesley, NADC
RM5598	NADC 5261; a1 2/5	60	15	15	54	183	183	89	61	ST-179	*butzleri*	Poultry	Unknown		I. Wesley, NADC
RM4846	NADC 5693	60	15	15	54	122	183	89	61	ST-180	*butzleri*	Ground pork	US		I. Wesley, NADC

Strain	Other designation	*aspA*	*atpA*	*glnA*	*gltA*	*glyA1*	*glyA2*	*pgm*	*tkt*	ST	Species	Source	Country (State)	Year	Depositor[a]/Reference[b]
RM4847	NADC 5694	60	15	15	54	122	183	89	61	ST-180	*butzleri*	Ground pork	US		I. Wesley, NADC
RM4841	NADC 5275	61	48	21	24	119	206	27	25	ST-181	*butzleri*	Chicken carcass	US		I. Wesley, NADC
RM4842	NADC 5277	61	48	21	24	119	206	27	25	ST-181	*butzleri*	Chicken carcass	US		I. Wesley, NADC
RM4851	NADC 9134	62	12	39	56	124	124	94	23	ST-182	*butzleri*	Turkey	US		I. Wesley, NADC
RM4852	NADC 9135	62	12	39	56	124	124	94	23	ST-182	*butzleri*	Turkey	US		I. Wesley, NADC
RM5209	Lagos 08	62	12	7	56	124	124	96	23	ST-183	*butzleri*	Poultry abattoir effluent	Nigeria	2000	On et al., 2004
RM5211	Lagos 11	63	51	21	24	126	209	27	25	ST-184	*butzleri*	Poultry abattoir effluent	Nigeria	2000	On et al., 2004
RM5216	Lagos 26	64	52	30	17	128	211	20	66	ST-185	*butzleri*	Poultry abattoir effluent	Nigeria	2000	On et al., 2004
RM5217	Lagos 33	65	53	26	57	137	137	97	67	ST-186	*butzleri*	Poultry abattoir effluent	Nigeria	2000	On et al., 2004
RM5219	Lagos 35	66	12	40	15	130	212	98	58	ST-187	*butzleri*	Poultry abattoir effluent	Nigeria	2000	On et al., 2004
RM5221	MEHMET BEYRON	67	13	2	58	87	87	99	68	ST-188	*butzleri*	Human	Turkey	2003	S. On
RM5223	AYDIN190370	67	13	2	58	87	87	99	68	ST-188	*butzleri*	Human	Turkey	2003	S. On
RM5222	ALPEREN KARACA	68	54	2	59	131	213	100	69	ST-189	*butzleri*	Human	Turkey	2003	S. On
RM5229	Kafkas 45	69	2	11	23	135	214	103	70	ST-190	*butzleri*	Chicken carcass	Turkey	2000	On et al., 2004
RM5237	BU15F	70	56	29	19	120	120	105	6	ST-191	*butzleri*	Poultry	UK	1995	S. On
RM5234	DVI DU14	71	25	7	2	138	215	106	2	ST-192	*butzleri*	Duck	Denmark	2000	On et al., 2004
RM5239	SX-347	71	25	7	2	102	215	106	2	ST-193	*butzleri*	Ovine feces	Sweden	1999	On et al., 2004
RM5240	SX-460	71	25	7	2	102	215	106	2	ST-193	*butzleri*	Ovine feces	Sweden	1999	On et al., 2004
RM5241	SX-457	71	25	7	2	102	215	106	2	ST-193	*butzleri*	Ovine feces	Sweden	1999	On et al., 2004
RM5242	SX-735	72	30	43	20	141	217	108	30	ST-194	*butzleri*	Ovine feces	Sweden	1999	On et al., 2004
RM5522	CDC D2892; 02834	73	2	11	44	146	146	111	40	ST-195	*butzleri*	Swine; stillborn piglet	N. Ireland	**(c)**	Kiehlbauch et al, 1991
RM5524	CDC D2885; 04955	73	2	11	44	146	146	111	40	ST-195	*butzleri*	Aborted swine fetus	N. Ireland	**(c)**	Kiehlbauch et al, 1991
R-700		73	12	1	65	188	220	10	9	ST-196	*butzleri*	Human	Germany		K. Houf
RM5525	CDC D2893; 02937	74	8	1	60	147	148	112	73	ST-197	*butzleri*	Swine; thoracic fluid	Ireland	**(c)**	Kiehlbauch et al, 1991
RM5526	CDC D2894; 02741	74	8	1	60	147	148	112	73	ST-197	*butzleri*	Aborted bovine fetus	Ireland	**(c)**	Kiehlbauch et al, 1991
RM5527	CDC D0232	75	25	30	26	65	149	26	74	ST-198	*butzleri*	Human; mitral valve	US (GA)	**(c)**	Kiehlbauch et al, 1991
RM5532	CDC D2451	76	61	26	61	155	155	115	77	ST-199	*butzleri*	Human; stool	US (IA)	**(c)**	Kiehlbauch et al, 1991
RM5534	CDC D2568	77	10	24	17	19	19	117	20	ST-200	*butzleri*	Human; blood	US (FL)	**(c)**	Kiehlbauch et al, 1991
RM5536	CDC D2630	78	64	47	48	157	157	97	80	ST-201	*butzleri*	Human; stool	US (WA)	**(c)**	Kiehlbauch et al, 1991
RM5541	CDC D2901	79	66	11	22	163	163	119	83	ST-202	*butzleri*	Human; stool	US (CO)	**(c)**	Kiehlbauch et al, 1991
LMG 15577		80	4	9	12	120	120	126	4	ST-203	*butzleri*	Adult diarrheal stool	Netherlands	1994	
RM5559	CDC D2685	80	67	49	25	171	172	7	26	ST-204	*butzleri*	Human; peritoneal fluid	US (CA)	**(c)**	Kiehlbauch et al, 1991
RM5594	NADC 4669; 202	81	69	26	66	182	182	124	86	ST-205	*butzleri*	Swine	Unknown		I. Wesley, NADC
RM5516	NADC 3156; 11780	37	5	11	30	120	127	16	72	ST-259	*butzleri*	Swine	Unknown		I. Wesley, NADC
RM4850	NADC 6830	14	49	26	55	144	144	92	13	ST-267	*butzleri*	Horse	US		I. Wesley, NADC
14166	STF-23	23	21	11	23	32	32	26	143	ST-272	*butzleri*	Fresh chicken	Thailand		C. Mason, AFRIMS
14130	622H-2004	82	78	59	67	223	ND	133	130	ST-206	*cryaerophilus*	Human; stool	France	2004	F. Megraud
RM5340	LMG 6622; CCUG 12018	83	70	52	68	249	249	134	97	ST-207	*cryaerophilus 1*	Aborted swine fetus; kidney	N. Ireland		Vandamme et al., 1992
14132	CCUG 12020	84	71	60	69	225	299	135	92	ST-208	*cryaerophilus 1*	Aborted swine fetus; eye	N. Ireland	1982	Vandamme et al., 1992
RM1582	CCUG 17801[T]	85	72	53	70	248	248	128	93	ST-209	*cryaerophilus 1*	Aborted bovine fetus; brain	N. Ireland	1985	Vandamme et al., 1992
RM6744	NADC 3577; 2601	85	72	53	70	248	248	128	93	ST-209	*cryaerophilus*	Bovine	Ireland		I. Wesley, NADC
RM4604	NADC 3256	86	79	61	71	265	21	136	98	ST-210	*cryaerophilus*	Human	Unknown		I. Wesley, NADC
RM4612	NADC 4075	86	79	61	71	265	21	136	98	ST-210	*cryaerophilus*	Swine	Unknown		I. Wesley, NADC
RM4832	NADC 4081	86	79	61	71	265	21	136	98	ST-210	*cryaerophilus*	Swine	Unknown		I. Wesley, NADC
RM4833	NADC 4082	86	79	61	71	265	21	136	98	ST-210	*cryaerophilus*	Swine	Unknown		I. Wesley, NADC
RM5557	CDC D2610; ATCC 49615	86	79	61	71	265	21	136	98	ST-210	*cryaerophilus 2*	Human; blood	US (IL)		

Strain	Other designation	*aspA*	*atpA*	*glnA*	*gltA*	*glyA1*	*glyA2*	*pgm*	*tkt*	ST	Species	Source	Country (State)	Year	Depositor[a]/Reference[b]
RM4491	NADC 3014	88	81	63	72	230	230	138	100	ST-211	*cryaerophilus*	Swine	Unknown		I. Wesley, NADC
RM4598	NADC 2780	89	79	61	73	231	231	138	101	ST-212	*cryaerophilus*	Horse	Unknown		I. Wesley, NADC
RM4599	NADC 2781	89	79	61	73	231	231	138	101	ST-212	*cryaerophilus*	Horse	Unknown		I. Wesley, NADC
RM4600	NADC 3017	90	82	61	74	271	193	139	102	ST-213	*cryaerophilus*	Swine	Unknown		I. Wesley, NADC
RM5576	NADC 3047; 17466	90	82	61	74	271	193	139	102	ST-213	*cryaerophilus*	Swine fetus	Unknown		I. Wesley, NADC
RM4602	NADC 3100	91	81	64	75	233	203	140	103	ST-214	*cryaerophilus*	Swine	Unknown		I. Wesley, NADC
RM4605	NADC 3479	93	84	66	73	235	303	142	105	ST-215	*cryaerophilus*	Swine	Unknown		I. Wesley, NADC
RM4614	S-1-1	95	87	69	78	238	234	145	107	ST-216	*cryaerophilus*	Poultry	US		M. Englen, USDA
RM4616	S-4-1	96	78	70	72	252	252	146	108	ST-217	*cryaerophilus*	Poultry	US		M. Englen, USDA
RM4617	S-3-1	97	88	71	73	239	239	147	109	ST-218	*cryaerophilus*	Poultry	US		M. Englen, USDA
RM4622	C-2-1	98	89	83	104	240	243	148	110	ST-219	*cryaerophilus*	Poultry	US		M. Englen, USDA
RM4826	NADC 3578	99	73	54	79	250	251	129	94	ST-220	*cryaerophilus*	Bovine	Unknown		I. Wesley, NADC
RM5341	LMG 9905	99	73	54	79	250	251	129	94	ST-220	*cryaerophilus 1*	Aborted bovine fetus; kidney	N. Ireland	1990	Vandamme et al., 1992
RM5585	NADC 3592; 2912	99	73	58	79	281	281	129	94	ST-221	*cryaerophilus*	Swine	Ireland		I. Wesley, NADC
RM4827	NADC 3588	100	74	90	80	241	242	149	111	ST-222	*cryaerophilus*	Swine	Unknown		I. Wesley, NADC
RM5342	CCUG 17802	100	74	90	80	252	242	149	111	ST-223	*cryaerophilus 2*	Aborted bovine fetus	N. Ireland	1985	Vandamme et al., 1992
RM4829	NADC 3598	102	71	73	82	244	245	151	113	ST-224	*cryaerophilus*	Bovine	Unknown		I. Wesley, NADC
RM4831	NADC 3603	102	71	73	82	244	245	151	113	ST-224	*cryaerophilus*	Bovine	Unknown		I. Wesley, NADC
RM5344		103	91	75	72	254	306	153	114	ST-225	*cryaerophilus*	Unknown	Unknown		W. Miller, USDA
RM5558	CDC D2639	104	92	76	84	266	272	154	115	ST-226	*cryaerophilus*	Human; stool	US (IA)	**(c)**	Kiehlbauch et al, 1991
RM5573	NADC 2706; 90-161	105	82	77	74	267	267	155	116	ST-227	*cryaerophilus*	Swine	Unknown		I. Wesley, NADC
RM5574	NADC 2734; 90-162	106	93	61	71	268	268	166	117	ST-228	*cryaerophilus*	Swine	Unknown		I. Wesley, NADC
RM5575	NADC 3046; 16238	107	81	62	85	269	274	156	118	ST-229	*cryaerophilus*	Swine fetus	Unknown		I. Wesley, NADC
RM5577	NADC 3155; 4166	108	94	78	71	273	285	157	119	ST-230	*cryaerophilus*	Swine	Unknown		I. Wesley, NADC
RM5578	NADC 3580; 2736	109	75	55	86	275	275	130	95	ST-231	*cryaerophilus*	Bovine	Ireland		I. Wesley, NADC
RM5583	NADC 3586; 2789	112	76	57	89	279	312	132	96	ST-232	*cryaerophilus*	Bovine	Ireland		I. Wesley, NADC
AC10		113	71	82	95	291	ND	165	127	ST-233	*cryaerophilus*	Bovine	Belgium		K. Houf
RM5584	NADC 3591; 2931	113	77	79	90	280	280	159	121	ST-234	*cryaerophilus*	Swine	Ireland		I. Wesley, NADC
RM5586	NADC 3732; 12934 Kid A sm	114	88	76	91	284	313	160	122	ST-235	*cryaerophilus*	Swine	US (IA)		I. Wesley, NADC
RM5589	NADC 4002; 2749-22	114	88	76	91	284	313	160	122	ST-235	*cryaerophilus*	Swine	Unknown		I. Wesley, NADC
RM5587	NADC 3738; 23575 B SC	115	93	80	92	282	282	161	123	ST-236	*cryaerophilus*	Swine	US (IA)		I. Wesley, NADC
RM5588	NADC 3873; 3326	116	95	99	93	289	290	162	124	ST-237	*cryaerophilus*	Swine	US (IA)		I. Wesley, NADC
RM5601	NADC 3876; 5069	116	95	99	93	289	290	162	124	ST-237	*cryaerophilus*	Swine	Unknown		I. Wesley, NADC
RM5602	NADC 4006; 5069 Kid B	116	95	99	93	289	290	162	124	ST-237	*cryaerophilus*	Swine	Unknown		I. Wesley, NADC
RM5599	NADC 3739; 24095 A SC	117	92	81	94	287	314	164	126	ST-238	*cryaerophilus*	Swine	Unknown		I. Wesley, NADC
RM4608	NADC 3482	125	86	68	77	237	237	144	106	ST-239	*cryaerophilus*	Swine	Unknown		I. Wesley, NADC
RM4490	NADC 3012	87	80	62	72	229	229	137	99	ST-264	*cryaerophilus*	Swine	Unknown		I. Wesley, NADC
RM4603	NADC 3144	92	83	65	76	232	228	141	104	ST-265	*cryaerophilus*	Swine	Unknown		I. Wesley, NADC
RM4828	NADC 3596	101	90	72	81	270	246	150	112	ST-266	*cryaerophilus*	Bovine	Unknown		I. Wesley, NADC
RM5343	DVI DU13	138	79	74	83	253	305	152	128	ST-268	*cryaerophilus 2*	Duck cloaca	Denmark	2000	On et al., 2003
RM5580	NADC 3576; 2602	110	85	67	87	276	277	158	120	ST-269	*cryaerophilus*	Swine	Ireland		I. Wesley, NADC
RM5582	NADC 3584; 2831	111	76	56	88	278	311	131	94	ST-270	*cryaerophilus*	Swine	Ireland		I. Wesley, NADC
RM5590	NADC 4623; 108	108	93	65	71	285	286	163	125	ST-271	*cryaerophilus*	Swine	Unknown		I. Wesley, NADC
RM5600	NADC 3870; 2749 (6/4)	108	93	65	71	285	286	163	125	ST-271	*cryaerophilus*	Swine	Unknown		I. Wesley, NADC
RM6739	NADC 3048; 16247	139	114	64	114	288	283	138	144	ST-274	*cryaerophilus*	Swine fetus	Unknown		I. Wesley, NADC
RM6740	NADC 3097; 90-26284	140	115	103	115	302	300	181	145	ST-275	*cryaerophilus*	Swine	Unknown		I. Wesley, NADC
RM6741	NADC 3098; 90-10146	141	116	61	116	315	315	182	98	ST-276	*cryaerophilus*	Swine	Unknown		I. Wesley, NADC
RM6742	NADC 3158; 17576 B2	142	117	61	117	317	316	183	146	ST-277	*cryaerophilus*	Swine	Unknown		I. Wesley, NADC

Strain	Other designation	*aspA*	*atpA*	*glnA*	*gltA*	*glyA1*	*glyA2*	*pgm*	*tkt*	ST	Species	Source	Country (State)	Year	Depositor[a]/Reference[b]
RM6743	NADC 3162; F22261	143	78	104	72	265	265	154	147	ST-278	*cryaerophilus*	Swine	Unknown		I. Wesley, NADC
RM6745	NADC 3582; 2737	144	78	105	74	318	318	184	148	ST-279	*cryaerophilus*	Bovine	Ireland		I. Wesley, NADC
RM6746	NADC 3585; 2782	102	118	60	69	320	319	185	149	ST-280	*cryaerophilus*	Swine	Ireland		I. Wesley, NADC
RM6747	NADC 3587; 2781	145	71	106	80	322	321	186	150	ST-281	*cryaerophilus*	Swine	Ireland		I. Wesley, NADC
RM6748	NADC 3595; 2778	110	85	67	87	324	323	158	120	ST-282	*cryaerophilus*	Swine	Ireland		I. Wesley, NADC
RM6749	NADC 3597; 2752	101	119	107	118	326	325	187	97	ST-283	*cryaerophilus*	Swine	Ireland		I. Wesley, NADC
RM6750	NADC 3599; 2749	146	120	108	85	328	327	188	151	ST-284	*cryaerophilus*	Bovine	Ireland		I. Wesley, NADC
RM6751	NADC 3600; 2770	110	85	67	87	330	329	158	120	ST-285	*cryaerophilus*	Bovine	Ireland		I. Wesley, NADC
RM6752	NADC 3601; 2796	147	121	109	81	332	331	189	152	ST-286	*cryaerophilus*	Swine	Ireland		I. Wesley, NADC
RM6753	NADC 3652; 23575 A Kid	93	84	110	119	334	333	190	153	ST-287	*cryaerophilus*	Swine	Unknown		I. Wesley, NADC
RM6754	NADC 3730; 12310 Kid A	148	78	111	74	336	335	191	154	ST-288	*cryaerophilus*	Swine	Unknown		I. Wesley, NADC
RM6755	NADC 4005; 3161-22	140	115	70	115	302	300	181	155	ST-289	*cryaerophilus*	Swine	Unknown		I. Wesley, NADC
RM6757	NADC 5269	149	122	112	120	338	337	192	156	ST-290	*cryaerophilus*	Turkey	US (IA)		I. Wesley, NADC
RM4607	NADC 3481	94	85	67	121	236	236	143	129	ST-291	*cryaerophilus*	Swine	Unknown		I. Wesley, NADC
LMG 10238		73	104	1	101	295	ND	171	137	ST-243	*skirrowii*	Calf with hemorrhagic colitis	Canada	1990	Vandamme et al., 1992
14127		122	99	87	101	226	222	169	132	ST-244	*skirrowii*	Pig	N. Ireland		
RM3223	ATCC 51400, LMG 8538	122	100	87	102	222	222	169	132	ST-245	*skirrowii*	Bovine	Unknown	1979	Vandamme et al., 1992
RM5406	BU 30CC 2B1	126	102	92	105	259	307	171	134	ST-246	*skirrowii*	Chicken carcass	UK		On et al., 2003
RM5409	BU 30CC 8B1	126	102	92	105	259	307	171	134	ST-246	*skirrowii*	Chicken carcass	UK		On et al., 2003
RM5407	DVI DU6CL	127	103	93	106	257	257	172	135	ST-247	*skirrowii*	Duck cloaca	Denmark	2000	On et al., 2003
RM5408	DVI DU1CL	128	104	94	101	258	258	169	139	ST-248	*skirrowii*	Duck cloaca	Denmark	2000	On et al., 2003
RM5413	16459	129	106	96	101	262	262	174	134	ST-249	*skirrowii*	Pig abortion	Denmark	1996	On et al., 2003
RM5424	16746-2	130	107	97	108	263	310	178	132	ST-250	*skirrowii*	Pig abortion	Denmark	1996	On et al., 2003
LMG 11075		131	108	100	101	296	ND	175	132	ST-251	*skirrowii*	Bull, preputial fluid	Belgium	1991	Vandamme et al., 1992
LMG 14983	CDC D4238	132	109	101	109	297	297	176	138	ST-252	*skirrowii*	Bull, preputial washing	US (NM)	1994	
LMG 9911		133	110	102	101	298	298	177	137	ST-253	*skirrowii*	Aborted swine fetus	N. Ireland	1990	Vandamme et al., 1992
RM5410	16854-1	134	105	95	107	260	308	173	136	ST-254	*skirrowii*	Pig abortion	Denmark		On et al., 2003
RM3222	NADC 3524, CCUG 10374[T]	122	100	87	101	226	222	169	132	ST-260	*skirrowii*	Lamb feces	UK	1980	Vandamme et al., 1992
RM3274	LMG 10234	123	101	88	103	227	301	169	132	ST-261	*skirrowii*	Aborted swine fetus	Canada	1990	Vandamme et al., 1992
RM5347	DVI DU3CL1	135	111	91	110	255	256	179	142	ST-255	*thereius*	Duck cloaca	Denmark	2000	On et al., 2003
RM5348	16389; LMG 24486[T]	135	111	91	111	255	256	179	142	ST-256	*thereius*	Pig abortion	Denmark	1998	On et al., 2003
RM5412	16695-3; LMG 24487	136	112	95	112	261	309	180	141	ST-257	*thereius*	Pig abortion	Denmark	1998	On et al., 2003
RM5517	NADC 3159; 26819	137	113	98	113	264	264	180	141	ST-262	*thereius*	Swine	Unknown		I. Wesley, NADC
RM5243	KH1	119	96	84	97	247	304	167	131	ST-240	*cibarius*	Chicken; broiler skin	Belgium	2002	K. Houf
RM5244	LMG 21996[T]	119	96	84	97	247	247	167	131	ST-240	*cibarius*	Chicken; broiler skin	Belgium	2002	Houf et al., 2005
RM5245	LMG 21997	119	96	84	97	247	304	167	131	ST-240	*cibarius*	Chicken; broiler skin	Belgium	2002	Houf et al., 2005
RM5246	KH4	119	96	84	97	247	304	167	131	ST-240	*cibarius*	Chicken; broiler skin	Belgium	2002	K. Houf
RM5248	LMG 21998	119	96	84	98	247	247	168	90	ST-241	*cibarius*	Chicken; broiler skin	Belgium	2002	Houf et al., 2005
CIB743		119	96	84	99	294	247	168	91	ST-242	*cibarius*	Chicken; broiler skin	Belgium	2002	K. Houf
CIB747		119	96	84	99	294	304	167	91	ST-263	*cibarius*	Chicken; broiler skin	Belgium	2002	K. Houf
CIB755		119	96	84	97	247	247	167	157	ST-273	*cibarius*	Chicken; broiler skin	Belgium	2002	K. Houf

Cryaerophilus alleles that represent the minor clade at the *atp*, *gln*, *pgm* and *tkt* loci are shaded in orange. *glyA* genes with different alleles at each locus are shaded in gray. *glyA2* alleles are included in

a. NADC: National Animal Disease Center, Ames, IA; AFRIMS: Armed Forces Research Institute of Medical Sciences, Bangkok, Thailand

b. Kiehlbauch et al., 1991: Kiehlbauch, JA et al. "*Campylobacter butzleri* sp. nov. isolated from humans and animals with diarrheal illness." J Clin Microbiol. 1991. **29**:376-85.

On et al., 2003: On, SL et al., "Differentiation of *Arcobacter* species by numerical analysis of AFLP profiles and description of a novel *Arcobacter* from pig abortions and turkey faeces." J Appl Microbiol. 2003. **95**:1096-105.

On et al., 2004: On, SL et al., "Genotyping and genetic diversity of *Arcobacter butzleri* by amplified fragment length polymorphism (AFLP) analysis." Lett Appl Microbiol. 2004. **39**:347-52.

Vandamme et al., 1992: Vandamme, P et al., "Polyphasic taxonomic study of the emended genus *Arcobacter* with *Arcobacter butzleri* comb. nov. and *Arcobacter skirrowii* sp. nov., an aerotolerant bacterium isolated from veterinary specimens." Int J Syst Bacteriol. 1992. **42**:344-56.

c. Strains from Kiehlbauch et al., 1991 were isolated between 1975 and 1989.
